# Uptake and leakage rates differentially shape community arrangement and composition of microbial consortia

**DOI:** 10.1093/ismejo/wraf122

**Published:** 2025-06-13

**Authors:** Estelle Pignon, Gábor Holló, Théodora Steiner, Simon van Vliet, Yolanda Schaerli

**Affiliations:** Department of Fundamental Microbiology, University of Lausanne, 1015 Lausanne, Switzerland; Department of Fundamental Microbiology, University of Lausanne, 1015 Lausanne, Switzerland; Department of Fundamental Microbiology, University of Lausanne, 1015 Lausanne, Switzerland; Department of Fundamental Microbiology, University of Lausanne, 1015 Lausanne, Switzerland; Biozentrum, University of Basel, 4056 Basel, Switzerland; Department of Fundamental Microbiology, University of Lausanne, 1015 Lausanne, Switzerland

**Keywords:** synthetic microbial consortium, engineered microbial community, spatial arrangement, auxotrophies, microbial interactions, amino acid exchange

## Abstract

Bacteria often grow as communities in intricate spatial arrangements on surfaces and interact with each other through the local exchange of diffusible molecules. Yet, our understanding of how these metabolite exchanges shape the properties of the communities remains limited. Here, we study synthetic communities of *Escherichia coli* amino acid auxotrophs interacting through the obligate exchange of amino acids. We genetically engineer these strains to alter their amino acid leakage and uptake abilities. We then characterize the spatial arrangement and composition of the communities when grown on a surface and compare these to qualitative predictions of a previously developed analytical model for cells growing in two dimensions. Our experiments provide empirical validation of the model’s central hypothesis: higher uptake rates reduce sector widths and promote mixing, while increased leakage rate of an amino acid increases the frequency of the strain benefiting from this amino acid. We thus extend the relevance of this simplified model to more complex, 3D systems, while also identifying its limitations. Our findings provide critical insights into microbial community dynamics and establish a predictive framework for designing and engineering microbial consortia.

## Graphical Abstract

**Graphical Abstract:**
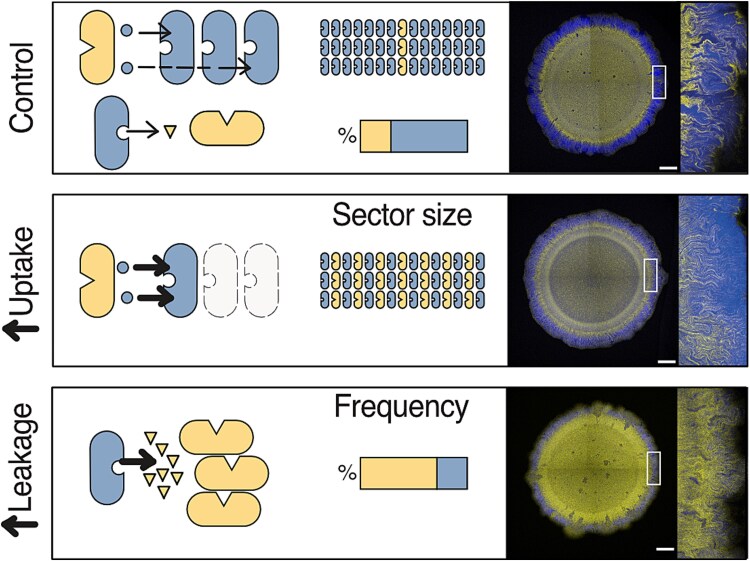

## Introduction

Microbial communities are ubiquitous on Earth and often grow on surfaces, for example in the form of biofilms [1]. Growth of surface-associated microbes is dependent on the physio-chemical properties of their local environment such as gradients of glucose, pH, viscosity or oxygen that influence the spatial arrangement of microbial communities [2–6]. In return, microbes actively change their environment as well, by consuming and releasing metabolites [7–9] or changing the local chemical properties [10]. Finally, microbes interact with each other, often through the exchange of diffusible molecules [11, 12]. Microbes leak a large diversity of metabolites [13], and at the same time, many microorganisms rely on the uptake of metabolites released by other community members. Indeed, many microbes are auxotrophic for certain compounds, meaning they are unable to synthesize a particular organic compound required for their growth [14]. This leads to diffusion-dominated interactions taking place in highly structured communities, making the microbial arrangement in space a crucial parameter for access to nutrients or the exchange of metabolites. Among the exchanged molecules, amino acids are highly prevalent [11, 14, 15] and amino acid can be found in a variety of environments, such as the mammalian gut [16, 17], the plant leaf [18] and even in oil reservoirs [19]. Amino acid auxotrophies have been shown to create strong interdependencies, which affect the dynamics of community compositions, carbon and energy fluxes, and host-microbiome interactions [20, 21]. They also promote high species diversity, drug tolerance, evenness and robustness of the consortium [22–24].

To study metabolite exchanges and the organization of spatially structured microbial communities, range expansion assays are a powerful approach [25–27]. They consist of inoculating either a single strain or a community, often fluorescently labelled, on an agar plate. The cells then expand radially, enabling the observation of spatial pattern formation. In addition, external parameters such as temperature, carbon sources, or other molecules can easily be modified. This setup enables one to study and understand in details the effect of a multitude of parameters on microbial communities such as mutualism and competition [28–30], trophic dependencies [6], short- or long-range weapons [31, 32], cellular growth rates [33, 34], or cell motility [35, 36].

**Figure 1:**
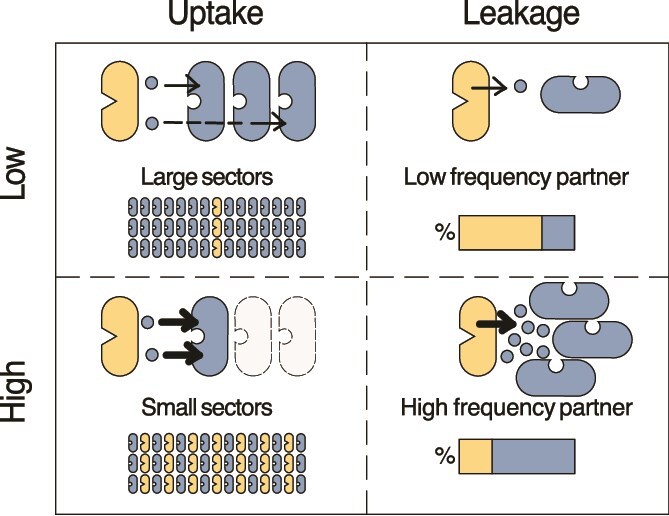
Key parameters predicted to determine community properties. Visual representation of cells exchanging amino acids (the amino acids are represented by small circles). Uptake: cells with a low uptake rate let a fraction of the exchanged metabolite to diffuse past them, enabling cells to grow further away from their partner and form patches of bigger size compared to cases with high uptake. Leakage: a low leakage rate leads to a low frequency of the partner cells and a high leakage rate to a high frequency.

Despite these recent advances, we still do not fully understand how metabolite exchanges between microbes determine the properties of communities, such as their composition and spatial arrangement. To address this question, Dal Co and colleagues studied a synthetic community of two auxotrophic *Escherichia coli* strains [37] that engage in reciprocal amino acid exchange. Each strain is unable to produce one essential amino acid (either tryptophan or proline), but they can grow together in a co-culture by exchanging these metabolites. The authors measured single-cell growth rates in a microfluidic device and concluded that the bacteria only interact with nearby cells. In a follow-up study, van Vliet and colleagues developed a mathematical framework [38] that derives community-level properties from the interactions at the local scale. In particular, the authors postulated that a few key biophysical parameters are sufficient to predict the arrangement and composition of the community. Those parameters are the cell density, as well as the leakage and uptake rates of the exchanged metabolites (i.e. amino acids in this case). In a dense range expansion community, the spatial arrangement and composition are thus predicted to be determined mostly by the uptake and leakage rate of the amino acids. In brief, the model predicts that increasing a strain’s uptake rate, decreases the size of the patches it forms, without strongly affecting its frequency. At first glance, this may seem counter-intuitive, as increased uptake should promote faster growth of the affected strain, thus leading to an expected increase in frequency. However, a high uptake rate also lowers the diffusion range of the exchanged molecule. This restricts cells with a high uptake rate to thrive only in close proximity to their partner, leading to smaller patches than strains with lower uptake rates. At the same time, higher uptake rates provide only a very limited growth benefit, as most cells are limited by the supply of amino acids and not by their uptake. As a result, uptake rates are predicted to have a strong influence on spatial patterning, but only a weak effect on community composition. In contrast, the model predicts that leakage rates are the main determining factor of the community composition, as they determine the relative supply of the growth limiting amino acids. Therefore, the strain that consumes the molecule with increased leakage rate is predicted to increase in frequency [38] (Fig. 1).

In this study, we rigorously tested whether these qualitative predictions hold in a realistic, three-dimensional biological system. Using targeted genetic engineering, we manipulated key biophysical parameters—namely, uptake and leakage rates—to directly investigate their effects on spatial organization and community composition. Rather than using constrained microfluidic chambers, we chose a larger, more complex experimental setup by performing range expansion experiments on agar plates, allowing a co-culture of proline and tryptophan *E. coli* auxotrophs to self-organize in an unconstrained spatial setting, reaching colony radii of up to 6 mm and thicknesses up to 65 μm (Figs S1, S2, S3 and S4). Through confocal microscopy and quantitative image analysis, we observed that increasing the proline uptake rate led to significantly smaller patches (or sectors) of proline auxotrophs. Further, we demonstrated that increasing the leakage rate of either amino acid altered community composition, favoring the auxotroph reliant on the overproduced amino acid. By simultaneously enhancing both uptake and leakage rates, we studied the combined effects of these changes on both spatial arrangement and community structure, demonstrating that these parameters can be leveraged to predictably shape microbial communities. To further deepen our insights, we compared our experimentally measured community composition also quantitatively with the predictions made by the mathematical model from Vliet et al. [38]. Overall, this simplistic model performed reasonably well in quantitatively capturing the observed strain frequencies, though it fell short in accurately predicting the results in certain cases. This study not only validates the model’s core hypothesis but also expands its applicability to complex, three-dimensional systems while identifying its limitations. By doing so, we deepen our understanding of microbial community dynamics in realistic environments.

## Material and methods

### Bacterial strains

All the experiments performed in this study were done using strains derived from *E. coli* MG1655 constitutively expressing sfGFP or mCherry and carrying the deletions $\Delta$*trpC* and $\Delta$*proC* (Addgene $\#$230037 and $\#$230036), respectively (kindly provided by A. Dal Co, [37, 39]). Details of all strains are listed in Table S1. All further gene deletions were performed with the prophage-lambda red recombination system as described previously [40]. The linear donor DNA fragment for the homologous recombination carried an antibiotic resistance gene and FRT sites. It was amplified from pKD3 or pKD4 by PCR (2× Phanta Max Master Mix, Vazyme) introducing extensions that are homologous to regions adjacent to the gene to be inactivated. After recombination, the antibiotic resistance gene was eliminated following the lamda red protocol. The primers used are listed in the Supplementary Table S2.

For the Tp strain (tryptophan overproduction) we introduced a *trpR* deletion. The linear donor DNA fragment was amplified from pKD3 using primers prEP167 and prEP168. The Pp strain (Proline overproduction) was generated by mutating a single nucleotide (A319G) in the *proB* gene [41]. To introduce this mutation, first, the *proBA* operon was deleted from the genome by recombination of a donor fragment amplified from pKD3 using the primers prEP197 and prEP198. We removed the antibiotic cassette following the lambda red protocol. Then we built pEP27, a plasmid derived from pKD4, harboring the FRT sites, the kanamycin resistance cassette and the *proBA* operon, including the single nucleotide mutation on proB. The plasmid backbone was obtained by amplifying pKD4 by PCR using the primers prEP177 and prEP200. *proBA* was amplified from the genome of MG1655 in two pieces: *proB* until the mutation, and *proB* from the mutation to *proA*, using prEP201 and prEP185, and prEP203 and prEP202. The 3 fragments were assembled using Gibson assembly (NEBuilder HiFi DNA Assembly Master Mix, NEB). To re-introduce the proBA operon (now with A319G) mutation on *proB*, we amplified the sequence FRT-Km-FRT-proBA (A319G) with the primers prEP205 and prEP250 and then used again the lambda red protocol [40].

The histidine auxotrophic strain was obtained by deletion of *hisD* using prEP207 and prEP208 (lambda red recombination amplifying the fragment from pKD3) and the histidine overproducer (Hp) was obtained by knocking-out the *hisL* gene, using prEP227 and prEP228 (lambda red recombination, amplifying the fragment from pKD3). The methionine auxotrophic strain was obtained by deletion of *metB* using prEP211 and prEP212 amplifying from pDK3.

### Plasmids

All strains in this study harbor either a plasmid with the anhydrotetracycline (aTc) inducible expression of the *putP* gene (pEP17) or an identical control plasmid (pEP28) lacking *putP*. These plasmids (Addgene $\#$229235 and $\#$229236) are presented in Supplementary Table S3. pEP17 was built using the backbone of pDSG360, which was a kind gift from Ingmar Riedel-Kruse (Addgene $\#$115601) [42]. The antibiotic resistance was changed from kanamycin to chloramphenicol with Gibson assembly. The primers prEP111 and prEP112 were used to amplify by PCR the relevant parts of pDSG360. Primers prEP113 and prEP114 were used to PCR amplify *putP* from the genome of MG1655. The plasmid was assembled by Gibson Assembly (NEBuilder HiFi DNA Assembly Master Mix, NEB). The control plasmid pEP28 was built from pEP17, by amplifying it without the *putP* gene with primers prEP206 and prEP111 and assembled with Gibson assembly (NEBuilder HiFi DNA Assembly Master Mix, NEB).

### Range expansions experiments

Range expansions were performed in six replicates. For each replicate, starter cultures were obtained by inoculating a single bacterial colony in 2 ml of lysogeny broth (LB) supplemented with chloramphenicol (50 μg/ml) and overnight incubation at 37°C with orbital shaking (200 rpm). The next morning, each culture was refreshed 1:100 (20 μl of starter culture in 2 ml of fresh LB medium) and grown for 4 h in identical incubation conditions. Cells were then washed twice with an equal volume of PBS (i.e. 500 μl of culture were washed with 500 μl of PBS) and strains were mixed at a 1:1 ratio at an optical density (OD_600_) of 2. Agar plates were prepared 24 h in advance to ensure reproducible humidity of the plates. From the co-culture, 1.5 μl were gently pipetted on M9 minimal medium plates (1.5% agar, 47.76 mM Na2HPO4, 22.04 mM KH2PO4, 8.56 mM NaCl, 18.69 mM NH4Cl, 1 mM MgSO4, 0.1 mM CaCl2 and 10 mM glucose) supplemented with chloramphenicol (50 μg/ml) and aTc (50 ng/ml) and where indicated with 50 mg/L of proline and 20 mg/L of tryptophan. Plates were placed in a 37°C incubator for 5 days. The range expansions were imaged with a Zeiss LSM 900 confocal microscope. sfGFP and mCherry were excited at 488 nm (0.8% intensity) and 561 nm (0.65% intensity) using a 45 μm pinhole, respectively. A 5× objective was used at 1.5× zoom, 15 Z stacks were taken for each image as well as 4 or 9 tiles depending on the size of the picture. The stitching was automatically carried out using the Zen Blue software.

### Image analysis

The steps of the image analysis are illustrated in Fig. S5. We measured the sector size and strain ratios for 6 different range expansions for each condition. We utilized the Bio-Formats toolbox to read the microscopic images in MATLAB [43]. As the colony grows, its height decreases with increasing radius, leading to varying intensity levels across different layers. To generate a single composite image of the colony, we extracted the maximum intensity value from each layer at every position, which corresponds to a projection of the colony surface onto the plane at the base of the colony.

Using this projection, we analysed the images to calculate specific colony measurements. First, we determined an intensity threshold for both colors using Otsu’s method [44]. The center and boundary of the colony were then identified using MATLAB’s *regionprops* function. Due to the slightly elongated, elliptical shape of the colonies, additional corrective steps were applied. After shifting the ellipsoid to the center of the coordinate system, we transformed the fitted ellipsoid into an equivalent circle using a simple linear transformation: ${M}_s\cdotp{M}_{\rho }$, where ${M}_{\rho }$ rotates the ellipsoid to align with the $x$-axis and ${M}_s$ scales it to a circular shape. The applied linear transformations are represented by the following matrices:


(1) \begin{equation*} {M}_s=\left[\begin{array}{cc}{\rho}_0/a& 0\\{}0& {\rho}_0/b\end{array}\right],\kern3.75em {M}_{\rho }=\left[\begin{array}{cc}\cos \phi & \sin \phi \\{}-\sin \phi & \cos \phi \end{array}\right], \end{equation*}

where ${\rho}_0$ is the equivalent radius of the circle, defined as ${\rho}_0=\left(a+b\right)/2$, with $a$ and $b$ representing half the lengths of the major and minor axes of the fitted ellipsoid, respectively. Additionally, $\phi$ denotes the orientation of the major axis of the ellipsoid, measured as the angle with respect to the $x$-axis. Although the colonies exhibit only slight ellipticity, this simple transformation enhances the accuracy and reliability of the method.

The volume of the initial inoculation droplet was the same in every experiment, so it was assumed that the radius of the inner ring was the same in every case as well: ${\rho}_c=1.89\cdotp{10}^3\ \mu m$. The *growth length* of the colony was defined as the difference between the radius of the colony and the radius of the inner ring: ${l}_{growth}= {\rho}_0-{\rho}_c$.

We transformed the colony into a polar coordinate system and analysed the 87.5%–95% radius range of the colony. The choice of this range is based on multiple factors: At the start of growth near the inner (inoculation) ring, we occasionally observed additional blue or yellow rings (Fig. S6A). We attribute this to dynamic changes in the community (Fig. S6B). As in this study, we wanted to focus on the steady state pattern (reached after approximately 2.5 days), we decided to analyse the images close to the edge of the colony. However, the colony boundaries are uneven, which limits the maximum usable radius. We used a relative range of the radius rather than a constant distance from the colony edge, because the properties of the colony can change during radial growth, and the final size of the colonies can vary across different conditions. Our method produces qualitatively consistent results even when using slightly different radius ranges (Fig. S7). The frequency of the yellow cells (${f}_{yellow}$) ($\Delta$*proC*) and the sector widths ($s$) was calculated in this radius range. The yellow frequency was determined based on the sum of the yellow (${F}_Y$) and blue (${F}_B$) fluorescent intensities along the ellipse: ${f}_{yellow}={F}_Y/\left({F}_Y+{F}_B\right)$.

The *sector width* ($s$) was defined as the shortest distance when the value of the autocorrelation function goes below a given threshold ($0.2$). This threshold was manually chosen, but we confirmed that all results are robust to its precise value (Fig. S8). The sector width was calculated along concentric ellipses within the selected radius range, using a step size of one pixel to project data points onto each ellipse. The distance between data points was determined using the arc length (${d}_{arc}$) of the ellipse:


(2) \begin{equation*} {d}_{arc}={\int}_0^{\Theta}\sqrt{a^2{\sin}^2\nu +{b}^2{\cos}^2\nu }\ d\nu, \end{equation*}

where $\Theta$ and $\nu$ are the polar angles in the transformed equivalent circle. The autocorrelation function was then computed for each concentric ellipse and averaged across the colonies.

One advantage of using the autocorrelation function is that it eliminates the need to categorize cells into distinct groups. Whereas compartmentalizing cell types can be effective when cells are clearly separated, this approach becomes challenging in well-mixed scenarios. Therefore, in this study, we employed this autocorrelation-based method, which is applicable in all cases, irrespective of the degree of mixing.

In some cases, we have found clear outliers. To reduce their impact, we employed a robust fitting method using bisquare weights with MATLAB’s “fit” function [45]. A constant function was fitted, points further from the fitted curve receive lower weights. The points significantly distant (that could not be expected by random chance), receive zero weight. We identified the points with zero bisquare weight as outliers and excluded them from our data.

### Model

The full model and its derivation were previously described in reference [38]; here we only summarize the most important assumptions and adaptations to this study.

#### Model assumptions

The model is based on the following main assumptions:

1. All cells take up amino acids with a constant uptake rate $u$. Amino acid concentrations are assumed to be low enough such that saturation of importers can be ignored.
2. Growth is limited by the amino acid for which a cell is auxotrophic. The growth rate $\mu$ follows Monod kinetics: $\mu =\frac{\mu_n\cdotp I}{I+1}$, where $I$ is the internal concentration measured in units of the Monod constant, and ${\mu}_n$ the maximum growth rate of the auxotroph in the presence of amino acids.
3. Producer cells maintain a constant internal concentration of ${I}_C$.
4. Cells externalize amino acids into the environment through passive diffusion across the cell membrane with leakage rate $l$.
5. Cells are homogeneously distributed through all of space. Molecules can only diffuse through the space between cells, and the effective diffusion rate is given by ${D}_{eff}=\frac{1-\rho}{1+\rho/2}D$, where $\rho$ is the cell density and $D$ the diffusion constant in solution [46].

These assumptions were based on the prevailing conditions in densely packed communities of auxotrophic cells, such as those found in 2D microfluidic growth chambers or in the core of 3D biofilms. However, we expect most of these assumptions to hold for the range expansion setup as well. One exception is the fifth assumption: in the range expansions setup, cells are coupled by molecules diffusing both through the colony and the agar. Accurately modelling these processes would significantly complicate the model and exceeds our objectives here. Instead, we made an additional simplifying assumption:

1. The agar surface underneath the colony acts as an absorbing boundary: i.e. amino acids that diffuse into the agar are lost from the system.

This assumption is justified by the agar’s substantially larger volume compared to the colony and the faster diffusion in agar (the diffusion coefficient in agar is nearly equivalent to that of water, whereas in the colony, it is significantly reduced due to cell crowding [46]). Consequently, we assume that cells interact exclusively through diffusion within the densely packed colony. Additionally, as some released amino acids are lost from the system, the leakage rate is adjusted to a lower effective value (see Supplementary Methods). Although reasonable, this assumption leads to an underestimation of the interaction range, as it neglects metabolic exchange mediated by the much faster diffusion through the agar beneath the colony.

The overproduction of amino acids could potentially affect two additional assumptions. Mutations that cause overproduction could disrupt internal homeostasis, triggering active externalization of these amino acids, thus violating the fourth assumption. Moreover, as amino acids are overproduced, their external concentrations can no longer be assumed to be low, meaning the first assumption—that uptake is non-saturated—could potentially also be violated. Consequently, we generally expect our model to perform worse for the overproducing communities.

#### Interaction range

Based on these assumptions, the range, ${R}_{int}$, over which amino acids are exchanged can be calculated analytically. We previously showed that this range is given by:


(3) \begin{equation*} {R}_{int}={r}_0\ln \left(\frac{\left(2{\tilde{l}}_n-\delta \right)\sqrt{4\delta +{\left({\tilde{l}}_n+\delta \right)}^2}-\delta \left(4+\delta \right)+{\tilde{l}}_n\left(2{\tilde{l}}_n-3\delta \right)}{2{\tilde{l}}_n\left({\tilde{l}}_n-\delta \right)-\delta}\right) \end{equation*}

where ${\tilde{l}}_n={l}_n/{\mu}_n$ is an auxotroph’s leakage rate relative to its growth rate and:


$$ {\displaystyle \begin{array}{c}{r}_0=\sqrt{\frac{D_{eff}}{\alpha \left({u}_n+{l}_n\right)}}\\{}\delta =\frac{l_p{I}_C}{2{\mu}_n}\cdotp \frac{2\sqrt{u_p+{l}_p}}{\sqrt{u_n+{l}_n}+\sqrt{u_p+{l}_p}}\cdotp \frac{u_n+{l}_n}{u_p+{l}_p}\end{array}} $$

where ${u}_p$ and ${l}_p$ are the uptake and leakage rates in the amino acid producing cells, ${u}_n$ and ${l}_n$ the rates in the non-producing auxotrophs, and $\alpha =\rho /\left(1-\rho \right)$ is the volume ratio of the intra- to extracellular space. Equation 3 above is equivalent to Equation 8 in reference [38], however, we did not use the simplifying assumption that leakage rates are low, as this is not necessarily the case for the communities containing overproducers. The biophysical approach—based on a reaction–diffusion model—used to derive the interaction above is similar to that used in previous works [28, 29, 47]. However, in all these studies the precise equations and results are slightly different as the reaction terms were adapted to the specific system of interest.

The interaction range characterizes the length scale over which cells can metabolically interact. This length scale places an upper limit on how far from the interface cells can grow and will thus affect the sector width, however sector width also depends on other non-metabolic factors such as the length scale of stochastic fluctuations at the interface between two sectors, the physics of cell growth, and the dynamics of radial expansion [27, 28].

As a result, there is a complex relationship between sector size and interaction range. Nonetheless, we expect that a decrease in interaction range—e.g. due to an increase in uptake rate—should also lead to a decrease in sector width.

#### Community composition

We previously showed that the equilibrium frequency of the community is given by:


(4) \begin{equation*} {f}_{\Delta P}=\frac{{\hat{\mu}}_{\Delta P}\cdotp \frac{N_{\Delta P}-2}{N_{\Delta P}}+\left(\frac{{\hat{\mu}}_{\Delta P}}{N_{\Delta P}}-\frac{{\hat{\mu}}_{\Delta T}}{N_{\Delta T}}\right)}{{\hat{\mu}}_{\Delta P}\cdotp \frac{N_{\Delta P}-2}{N_{\Delta P}}+{\hat{\mu}}_{\Delta T}\cdotp \frac{N_{\Delta T}-2}{N_{\Delta T}}} \end{equation*}

where ${f}_{\Delta P}$ is the final frequency of the proline auxotrophs. ${\hat{\mu}}_{\Delta P}$ and ${\hat{\mu}}_{\Delta T}$ in equation 4 are the maximum growth rates which the proline and tryptophan auxotrophs can reach when they are fully surrounded by amino acid producing cells. We previously showed that this maximum growth rate is given by:


(5) \begin{equation*} {\displaystyle \begin{array}{c}\hat{\mu}=\frac{\mu_n\hat{I}}{1+\hat{I}}\\{}\hat{I}=\frac{\left({u}_n+{l}_n\right)\hat{E}-{l}_n+\sqrt{{\left(\left({u}_n+{l}_n\right)\hat{E}+{l}_n\right)}^2+4\left({u}_n+{l}_n\right){\mu}_n\hat{E}}}{2\left({\mu}_n+{l}_n\right)}\\{}\hat{E}=\frac{l_p}{u_p+{l}_p}\cdotp{I}_C\end{array}} \end{equation*}

here $\hat{E}$ corresponds to the external concentration of the leaked amino acid in a region of space that is fully occupied by producer cells, and $\hat{I}$ is the corresponding internal concentration of that amino acid in a isolated auxotrophic cell in that a region (both measured in units of the Monod constant). Equation 5 is equivalent to Equation 7 in reference [38], however, we did not use the simplifying assumption that leakage rates are low.

Moreover, ${N}_{\Delta P}$ and ${N}_{\Delta T}$ in Equation 4 are the number of cells with which the proline and tryptophan auxotrophs interact, respectively. This can be calculated from the interaction range, using a simple geometric argument. We assume cells are cylinders with spherical caps with a total length $L$ and diameter $W$, and that they interact with all cells that are within distance ${R}_{int}$ from their cell surface (in three dimensions). The number of interaction partners is then given by:


(6) \begin{equation*} N=\frac{R_{int}\left({R}_{int}+W\right)\left(L-W\right)+4/3\left({\left({R}_{int}+W/2\right)}^3-{\left(W/2\right)}^3\right)}{\left(L-W\right){\left(W/2\right)}^2+4/3{\left(W/2\right)}^3}\cdotp \rho \end{equation*}

The composition of the community primarily depends on the leakage rates. Specifically, in communities where both strains have the same uptake and leakage rates (i.e. ${l}_p={l}_n$ and ${u}_p={u}_n$) the leakage rates fully determine which community member is more abundant: namely the one that depends on the amino acid that has the highest leakage flux (${l}_p{I}_C$). However, the exact frequency of the dominant community member is influenced by the uptake rate: Higher uptake rates lead to more local interactions, skewing the composition further in favor of the dominant community member. If strains differ in their uptake and leakage rates, the community composition depends on the exact values of these rates. Generally, leakage rates still have the strongest effect, however increasing the uptake rate in the auxotroph, relative to that of the producer (i.e. ${u}_n>{u}_p$) will increase its frequency to some extent.

In deriving Equation 4, we assumed that amino acids are always growth limiting. Under this assumption, the growth rate of a cell increases linearly with the frequency of producer cells within its interaction neighborhood, allowing the equilibrium frequency to be calculated analytically. However, this assumption might be violated in overproducing communities, where cells in close proximity to overproducing partners might no longer be growth-limited by amino acids.

#### Model parametrization

Model parameters are shown in Supplementary Table S4. Most parameters where obtained from literature, or from previous estimates from single-cell imaging experiments. However, no accurate estimates could be obtained for the effective leakage rates of proline and tryptophan, so these were estimated by fitting the model to the data. Moreover, we were unable to obtain an estimate for the increase in proline uptake rate conferred by the *putP* mutant. We therefore treated it as a free parameter and performed model predictions across a range of values, assuming a 10- to 100-fold increase in proline uptake.

The effective leakage rates of proline and tryptophan in the $\Delta$*trpC* and $\Delta$*proC* strains, respectively, were simultaneously fitted by minimizing the combined normalized Euclidean distance between the predicted and observed community composition and relative growth rate of the Ctr community. Specifically, we minimized ${\left(\frac{f_{pred}-{f}_{data}}{f_{data}}\right)}^2+{\left(\frac{r_{rel, pred}-{r}_{rel, data}}{r_{rel, data}}\right)}^2$, where ${f}_{pred}$ and ${f}_{data}$ are the predicted and mean observed frequency of $\Delta$*trpC* in the Ctr community and ${r}_{rel}$ is the growth of the auxotrophic community in the absence of amino acids relative to that in the presence of amino acids. The relative growth rate observed in the data, ${r}_{rel, data}$, was estimated as the ratio of the final colony diameters of the Ctr community in the absence of amino acids relative to that in the presence of amino acids. The value predicted by the model, ${r}_{rel, pred}$, is given by [38]:


$$ {\displaystyle \begin{array}{c}{r}_{rel, pred}=\frac{f_{\Delta P}\left(1-{f}_{\Delta P}\right)}{\mu_n}\left(\frac{N_{\Delta P}-2}{N_{\Delta P}-1}{\hat{\mu}}_{\Delta P}+\frac{N_{\Delta T}-2}{N_{\Delta T}-1}{\hat{\mu}}_{\Delta T}\right)\end{array}} $$

The effective leakage rates of proline in $\Delta$*trpC proB74* and of tryptophan in $\Delta$*proC*  $\Delta$*trpR* were fitted by minimizing the Euclidean distance between the predicted and observed community composition in the Pp and Tp communities, respectively. We used prior estimates that leakage rates are generally much lower (at least 1000× fold) than uptake rates [48], to constrain the range of values over which to perform the fit. Given that the proline and tryptophan synthesis pathways are largely independent, we assumed that *putP* overexpression did not affect the tryptophan leakage rate, and therefore applied the effective leakage rate fitted to the $\Delta$*proC* and $\Delta$*proC*  $\Delta$*trpR* strains to the $\Delta$*proC* + *putP* and $\Delta$*proC*  $\Delta$*trpR* + *putP* strains, respectively.

We assumed (assumption 3 above) that the internal amino acid concentration in producing strains (${I}_c$) is always the same, and that differences in externalization are due to differences in the leakage rate $l$. However, when $l<<u$ (which holds for all strains used here) the model only depends on the product of these two parameters $l\cdotp{I}_c$. By fitting the leakage rate we thus automatically compensate for any potential changes in the internal concentration caused by the genetic manipulation.

## Results

### Characterizing a synthetic community of two auxotrophs

**Figure 2:**
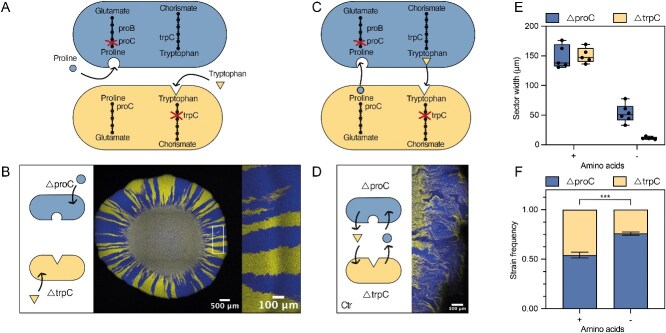
Co-culture of proline and tryptophan auxotrophs in the presence and absence of amino acids. (A) Schematic representing the relevant amino acid pathways of the strains used in this study. Knock-outs (proC and trpC) lead to proline or tryptophan auxotrophic strains, respectively. Here, proline and tryptophan were added to the medium. (B) Microscopy image of the range expansion. A representative image of one of the six biological replicates that were analysed is shown. Close-up on the edge of one of the expansions (marked with a white rectangle), at the radius where we analysed the community around the colony. (C) Schematic representation of the strains without addition of amino acids in the media. The amino acids are exchanged between the members. (D) Representative microscopy image of the edge of the range expansion without amino acids (control community, Ctr). (E) Quantification of the sector widths of both conditions. Box plots show the median value of the sector size of six biological replicates, error bars show min and max values. (F) Quantification of the frequencies of the two communities. Six biological replicates were analysed. The plot shows mean value and standard deviation. Student *t*-test, *** significant at *P*-value $<$ .0001.

We studied a synthetic community composed of two *E. coli* strains auxotrophic for amino acids (Fig. 2A). The first strain is a proline auxotroph ($\Delta$*proC*), which is unable to produce this amino acid due to the deletion of *proC* coding for an essential enzyme in the proline biosynthesis pathway. Similarly, the second strain ($\Delta$*trpC*) cannot synthesize tryptophan, because it contains a deletion of *trpC*, which catalyzes a step of the tryptophan biosynthesis pathway. Although the individual strains do not grow in minimal media, the two auxotrophs can grow as a co-culture. The cells naturally leak amino acids that diffuse into the medium and can be taken up through import systems [11, 49, 50].

To establish a baseline for our comparative analysis, we first characterized our co-culture in a defined minimal medium containing proline and tryptophan. In this environment, the strains do not rely on each other for growth but compete for the resources present in the medium. To study spatial organization and community composition during range expansion, we deposited a drop of the co-culture at a 1:1 ratio on an agar plate and let them grow outwards for a total of five days (Fig. 2B). We then imaged the range expansions by confocal microscopy (Fig. S1), capturing the community’s arrangement across all axes (x, y and z, Figs. S3 and S4). In all analyses, we only considered the edge of the expansion at 87.5-95% of the colony radius where the cells grew outwards (see Fig. S7, and Methods for details). We selected this region because this is where the community composition is constant (Fig. S6). To quantify the spatial arrangement, we measured the average sector size (Fig. 2E) of each member.

Finally, we determined the relative frequency of each strain by image analysis (Fig. 2F). In this initial control experiment with supplemented amino acids, both strains were equally abundant and the sectors did not differ in size. These results agree with our expectations, as the two strains have the same growth rates in this condition (Fig. S9). The pattern that we observed here—well-defined sectors and a clear segregation between the members—is typical of a range expansion of two strains competing for resources [27, 51–53].

To characterize the metabolite exchanges in our synthetic community of auxotrophs we studied its behavior in the absence of added amino acids (Fig. 2C), this is our control community (Ctr) for the rest of the results. We observed overall less growth (Fig. S2) and 3–14 times smaller sectors, i.e. much higher mixing between the two strains (Fig. 2D) compared to the condition where amino acids were supplemented. This was expected as now the cells can only grow in close proximity to their partner. Furthermore, the community exhibited asymmetry, with $\Delta$*proC* sectors being significantly wider (50.9$\pm$15.4 $\mu$m, median $\pm$ SD) compared to the $\Delta$*trpC* sectors (10.7$\pm$6.2 $\mu$m) (Fig. 2E). As a result, $\Delta$*proC* accounted for the majority of the population, comprising 75.8% of the community (Fig. 2F). The observed asymmetry in sector size and community composition agrees well with the previously observed patterns of the same community grown in microfluidic devices [37]. Specifically, the observed community composition in the range expansions (75.8%$\pm$0.1 of $\Delta$*proC*) is almost identical to that observed in the microfluidic chambers (75.5%$\pm$0.2 of $\Delta$*proC*) [37].

### Enhanced amino acid uptake results in smaller sector sizes for the strain with increased uptake

**Figure 3:**
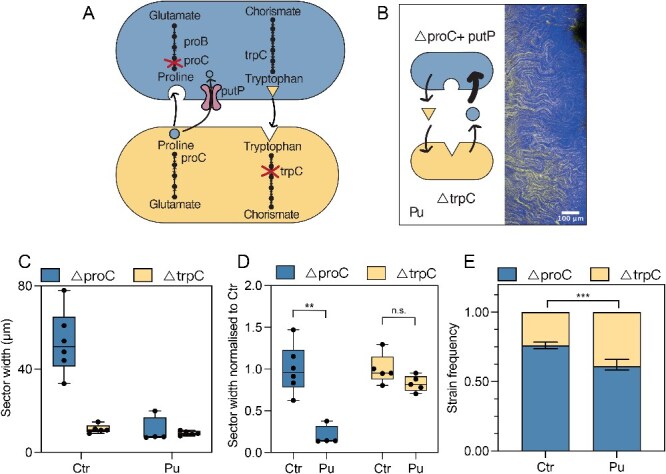
Increased proline uptake leads to smaller sectors of the proline auxotroph. Community with increased proline uptake (Pu) in the proline auxotroph. (A) Detailed view of the auxotroph community with proline importer (putP) overexpressed, leading to increased proline uptake. (B) Representative microscopy image of the range expansions. (C) Quantification of the sector widths in the proline uptake community (Pu) for the proline ($\varDelta$proC + putP) and the tryptophan ($\varDelta$trpC) auxotrophs. (D) Sector width normalized to median sector size of control. The box plot shows the median value of six biological replicates, error bars show min and max values. Wilcoxon test, ** significant at *P*-value $<$ .01. (E) Quantification of the frequencies of the two community members. Six biological replicates were analysed. The plot shows mean value and standard deviation student *t*-test, *** significant at *P*-value $<$ .001, n.s. at *P*-value = .158.

As outlined in the introduction (Fig. 1), the model predicted that increasing a strain’s uptake rate would lead to smaller sector widths, as the reduced diffusion range of the amino acid would limit the strain’s spatial expansion, with minimal impact on the sectors of the other strain and community composition. Strictly speaking, the model predicts the interaction range rather than the sector width. Although the precise relation between the metabolic interaction range and sector size is rather complex, generally it holds that decreasing the interaction range should lead to a decrease in sector size and we direct interested readers to the Methods section for a more detailed explanation. To test these qualitative predictions experimentally, we constructed a plasmid containing the *putP* gene, coding for a Na^+^/L-proline transporter, an active proline importer in *E. coli* [54, 55]. The overexpression of *putP* increases the proline uptake in the $\Delta$*proC* strain. In a range expansion of this $\Delta$*proC* + *putP* strain in co-culture with $\Delta$*trpC* (Proline uptake community, Pu), we observed 85.2% decrease in the size of $\Delta$*proC* (blue) sectors, whereas there is no statistically significant difference for the width of the $\Delta$*trpC* sectors (yellow) compared to the control community (Fig. 3C and D). Thus, the model’s qualitative prediction of a reduced sector width for the strain with the increased uptake rate ($\Delta$*proC* in the Pu community) was fully validated and by overexpressing *putP* we could confirm the importance of the uptake rates in shaping the spatial arrangement of the synthetic community.

For the effect on the community composition, the model predicted that the increase in uptake rate should cause the community composition to change only slightly, namely to skew further in favor of the majority type, i.e. in our case increasing the frequency of $\Delta$*proC*. However, we observed a decrease in $\Delta$*proC* frequencies (Fig. 3E). We also observed that this strain has a strongly reduced growth rate (80% growth rate reduction in liquid monoculture, Fig. S9). This is most likely caused by a burden coming from the overexpression of *putP*. According to the model, the basal growth rate of a strain has a large impact on its final frequency, so the decrease in $\Delta$*proC* frequency could be explained by this growth defect. Indeed, when this growth cost is taken into account, the model accurately predicts the decrease in $\Delta$*proC* frequency (Fig. S10). We will discuss these quantitative predictions in more detail later in the manuscript.

### Amino acid overproduction favors the auxotroph reliant on the overproduced amino acid

**Figure 4:**
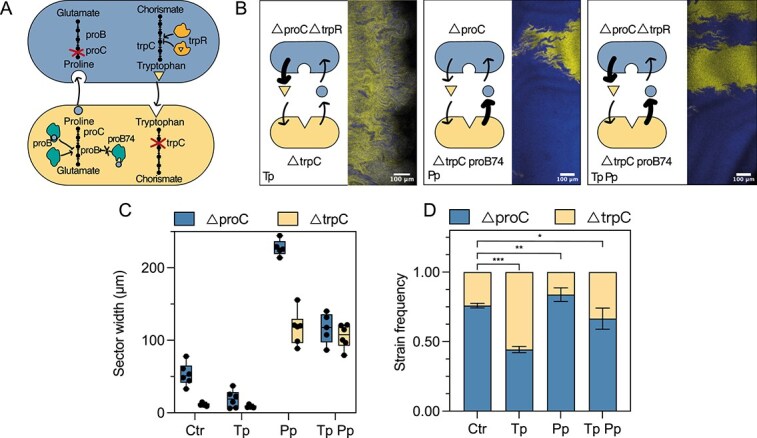
Amino acid overproduction leads to a change of community composition. Comparison between the initial community (Ctr) and communities with increased production of tryptophan (Tp), proline (Pp) or both (TpPp). (A) Detailed view of the metabolic pathways and modifications in the communities used in this assay. TrpR regulates the tryptophan pathway, a deletion of its gene leads to overproduction of tryptophan. proB regulates the proline pathway, and the mutation proB74 leads to proline overproduction. (B) Representative microscopy images of the range expansions. (C) Quantification of sector widths of each community for six biological replicates per condition, boxplot shows median and error bars show min and max values. (D) Quantification of community composition for six biological replicates. The plot shows mean value and standard deviation. Student *t*-test, * significant at *P*-value $<$ .01 and *** significant at *P*-value $<$ .0001.

The model also predicted that amino acid availability plays a crucial role in determining the frequencies of the community members by favoring the auxotroph dependent on the overproduced amino acid [38, 56]. This qualitative prediction led us to investigate the impact of increasing leakage rates. We constructed two strains that overproduce the amino acid required by their partner strain. We engineered a proline auxotroph overproducing tryptophan and a tryptophan auxotroph overproducing proline (Fig. 4A). The tryptophan overproduction was obtained by deleting *trpR*, a repressor of the tryptophan biosynthesis pathway [57]. The proline overproducer contains a point mutation (A319G) in the *proB* gene as this mutation (designated as *proB74*) was reported to lead to overproduction of proline in *E. coli* due to a loss of allosteric regulation [41].

To test the model’s prediction that community composition would change in response to the increased leakage caused by amino acid overproduction, we analysed the community composition of three overproducer communities: (i) Tp: tryptophan overproducer ($\Delta$*proC*  $\Delta$trpR) with $\Delta$*trpC*, (ii) Pp: proline overproducer ($\Delta$*trpC proB74*) with $\Delta$*proC* and (iii) Tp Pp: both tryptophan ($\Delta$*proC*  $\Delta$trpR) and proline overproducers ($\Delta$*trpC proB74*). Indeed as predicted, we observed an increase in the frequency of the strain benefiting from the overproduced amino acid (Fig. 4E): $\Delta$*trpC* increased in frequency when paired with the tryptophan overproducer in the Tp community and $\Delta$*proC* showed a higher frequency when paired with a proline overproducer in the Pp community. In conclusion, by overproducing the amino acids we demonstrated the importance of the leakage rates in determining the composition of the synthetic community, confirming the model’s qualitative prediction.

According to the model, increasing the leakage rate should only have a minor effect on the interaction range. Figure 4B shows the spatial pattern of the overproducer communities. As in the Ctr community, the $\Delta$*proC* strain formed consistently bigger sectors than the $\Delta$*trpC* strain. Yet, we observed much larger range expansions for the communities with the proline overproducer (Pp and Tp Pp) (Fig. S2). As a result, the absolute values of the sector sizes were much larger for those communities (Fig. 4C), a phenomenon not easily explained by the model. This discrepancy likely arises from the assumption in the model that amino acid concentrations remain low; however, amino acid overproduction violates this assumption. We will discuss this in more detail below.

### Effects of uptake and overproduction are cumulative

**Figure 5:**
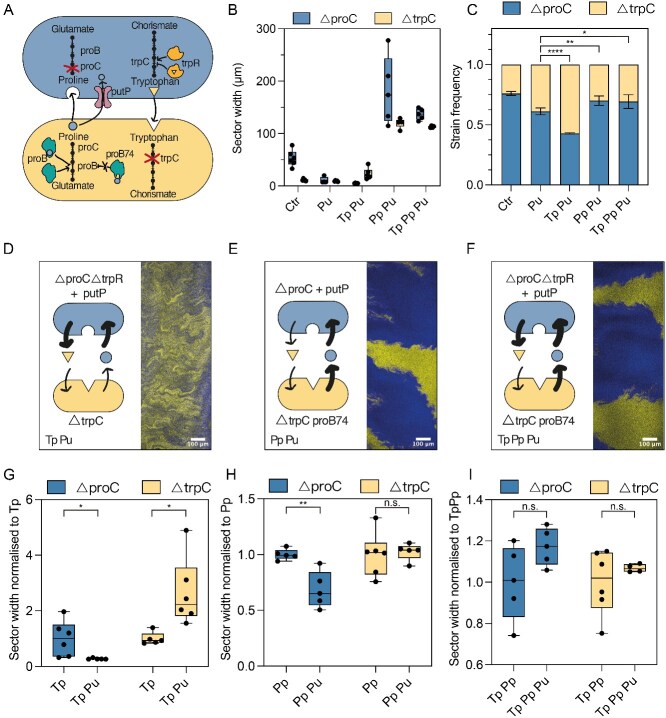
Combined effect of increased amino acid uptake and overproduction. Comparison between initial community (Ctr) and communities with tryptophan overproduction and increased proline uptake (Tp Pu), proline overproduction and increased proline uptake (Pp Pu), and both tryptophan and proline overproduction and increased proline uptake (Tp Pp Pu). (A) Detailed view of the metabolic pathways and modifications in the communities used in this assay. (B) Quantification of sector widths of each community. Box plots show the median of six biological replicates, error bars show min and max values. (C) Quantification of community composition. The plot shows mean value and standard deviation. Student *t*-test, * significant at *P*-value $<$ .05, ** significant at *P*-value $<$ .01, *** significant at *P*-value $<$ .001. (D–F) Representative microscopy images of the range expansions. (G–I) Sector sizes normalized to median sector size corresponding community without proline uptake. Wilcoxon test, * significant at *P*-value $<$ .05, ** at *P*-value $<$ .01, n.s. at *P*-value $>$ 0.1.

Having confirmed the individual effects of amino acid uptake and overproduction rates, we next investigated whether their predictable impacts persisted when both factors were combined. Our expectation was that increased proline uptake should lead to smaller $\Delta$*proC* sectors and a higher production of one amino acid should increase the frequency of the partner strain, independent of the strain background. We thus tested all the combinations of modifications that we had constructed. Namely, a community of the $\Delta$*proC* strain with tryptophan overproduction and increased proline uptake combined with the $\Delta$*trpC* strain (Tp Pu), a community of the $\Delta$*proC* strain with increased proline uptake combined with the $\Delta$*trpC* strain overproducing proline (Pp Pu), as well as a community of the $\Delta$*proC* strain with tryptophan overproduction and increased proline uptake combined with the $\Delta$*trpC* strain also exhibiting increased proline uptake (Tp Pp Pu) (Fig. 5A).

As we had observed previously, communities containing the proline overproducer strain grew more than the control community (Fig. S2), leading to overall bigger sectors (Fig. 5B). Although our model does not explain this increase in absolute sector size, we still expect its prediction to hold that—all else being equal—increasing uptake rate should reduce sector size. Consistent with this, we observed that increasing proline uptake rates reduced the sector size of the proline auxotroph in the Tp Pu and Pp Pu communities compared to their respective baseline communities (Tp, Fig. 5G; Pp, Fig. 5H). However, there was no significant change in the community in which both amino acids were overproduced (Tp Pp Pu vs. Tp Pp, Fig. 5I). We speculate that this may be because amino acids no longer limit the growth of either strain in this community.

For community composition, we analysed how introducing amino acid overproduction affected communities containing strains carrying the *putP* plasmid. Consistent with our expectations, we observed that increasing the production of an amino acid in one strain led to higher frequencies of the partner strain that depends on it (Fig. 5C). Specifically, there was a significant increase in the frequency of the $\Delta$*trpC* strain in the Tp Pu community compared to the Pu community, due to increased tryptophan production (Fig. 5C). Similarly, there was a significant increase in the frequency of the $\Delta$*proC* strain in the Pp Pu community compared to the Pu community, due to increased proline production (Fig. 5C). Finally, for the community with proline and tryptophan overproduction (Tp Pp Pu) we found that the proline auxotroph increased in frequency to 67% (Fig. 5C) compared to 60% in the Pu community. To summarize, we have shown that the combination of uptake and leakage rates is cumulative and predictably changes the community arrangement and composition accordingly.

### Quantitative agreement with the mathematical model

Our primary goal was to test the model’s qualitative predictions—that uptake and leakage rates predominantly influence a community’s spatial arrangement and composition, respectively—but we also wanted to assess its accuracy in quantitatively predicting community composition. To do this, we first needed to parametrize the model. Most parameters could be estimated from the literature. However, we could not obtain estimates for the effective amino acids leakage rates of the wild-type and overproducing strains; therefore, these were inferred by fitting the model to data from the Ctr, Tp, and Pp communities (Methods). Additionally, we were unable to estimate the increase in proline uptake rates conferred by *putP* overexpression. We thus ran our model over a range of values assuming a 10- to 100-fold increase.

**Figure 6:**
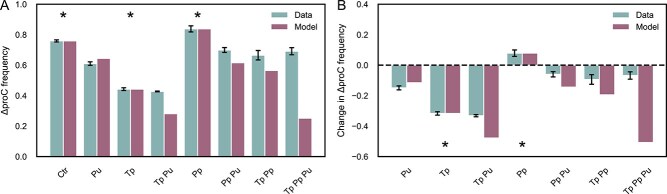
Comparison between model predictions and experimental data for strain frequencies. (A) Comparison between the observed (left bar) and predicted (right bar) frequency of $\varDelta$proC. (B) Observed (left bar) and predicted (right bar) changes in the frequency of $\varDelta$proC relative to the Ctr community. For the data, mean values and standard errors are shown. $\ast$ indicate data that was used to fit the effective leakage rate of tryptophan and proline in the control and overexpressing strains. Model predictions are shown assuming a 50-fold increase in proline uptake in the putP overexpressing strains, predictions are robust to changing this value (Fig. S10).

Before discussing the model’s *quantitative* accuracy, we first review its *qualitative* performance. Overall, the model accurately predicted the effects of changes in uptake and/or leakage rates on community composition, correctly capturing the direction of change for all communities (Fig. 6). These predictions are insensitive to the assumed fold-increase in proline uptake conferred by *putP* (Fig. S10). The model could explain the decline in the frequency of the $\Delta \mathrm{proC}+\mathrm{putP}$ strain: although $\mathrm{putP}$ overexpression should have moderately increased its frequency, the substantial growth cost fully accounts for the observed decrease. Moreover, the parameter inference suggests that the leakage rate of tryptophan increased more strongly than that of proline in the overproducing strains (34-fold increase in tryptophan leakage versus 6.6-fold increase in proline leakage; Table S4). This aligns with our observation that, in communities containing both tryptophan and proline overproducers (Tp Pp and Tp Pp Pu), the frequency of the $\Delta$*trpC* strain increased relative to the Ctr community, whereas the frequency of the $\Delta$*proC* decreased. (Figs 4D and 5C). The model was generally also able to predict qualitatively how changes in uptake rates affect changes in sector size: it correctly predicted that increasing proline uptake would decrease the sector size of $\Delta$*proC* without affecting the sector size of $\Delta$*trpC*, except for two cases ($\Delta$*proC* sector size in Tp Pp Pu and $\Delta$*trpC* sector size in Tp Pu, Fig. S11). The precise changes in sector size depend strongly on the assumed fold-increase in proline uptake conferred by *putP*, nevertheless the overall trends are robust to variation in its value.

Although our model performed well in capturing *qualitative* trends, its *quantitative* accuracy was more limited. It often overestimated the magnitude of changes in strain ratios, particularly in the community containing the proline overproducer (Pp, Fig. 6). Additionally, the model failed to predict the increase in absolute sector size caused by amino acid overproduction (Fig. S11). These discrepancies likely arise from violations of the model’s assumptions that amino acid concentrations are always low, causing it to fail in two ways. First, the equation for the equilibrium composition was derived under the assumption that amino acids are always growth-limiting. This assumption may not hold for cells growing near overproducing strains, potentially explaining the quantitative mismatch in predicted equilibrium frequencies. The largest deviations occurred in communities containing proline overproducers, consistent with the observation that the inferred proline leakage rate was higher than that of tryptophan (Table S4). Second, the equation for interaction range was derived assuming amino acid concentrations are low enough to ignore transporter saturation. This too may be violated in the vicinity of overproducers, leading to an underestimation of the interaction range in these communities. Further discrepancies may also stem from the fact that our model only predicts the metabolic interaction range, rather than directly forecasting sector widths, as observed in range expansion experiments. Previous studies have shown that the width of a colony sector is influenced by both the range over which metabolites are exchanged—placing an upper limit on the distance a cell can be from its partner strain supplying the growth-limiting nutrient—and by the physics of cell growth, which governs how quickly the boundary between sectors moves [28]. Although the relationship between the metabolic interaction range and sector size is complex, it generally holds that decreasing the interaction range leads to a reduction in sector size. Nevertheless, neglecting other factors affecting sector size, such as the physics of cell growth and movement and the dynamics of radial expansion [28] might contribute to the observed discrepancies. Whereas the model could, in principle, be extended to include saturating uptake and growth functions, as well as the effects of radial expansion, doing so would complicate the mathematical analysis, and we therefore leave such extensions for future work.

## Discussion

In this study, we described how two main parameters—amino acid uptake and leakage rates—determine a community’s spatial arrangement and composition (Fig. 1). The use of a simple synthetic community (Fig. 2), combined with genetic engineering of the strains, allowed us to modify these two variables independently. In agreement with the hypothesis previously proposed in a computational model [38], we experimentally demonstrated that increased amino acid uptake leads to smaller sectors, and thus a more mixed community, as the cells have to be close to each other to benefit from the shared molecule (Fig. 3). An increased amino acid leakage by one cell type leads to a higher frequency of its partner as the amino acid it is lacking is present at a higher concentration (Fig. 4). Changes of the two parameters can be combined to control spatial arrangement and composition at the same time (Fig. 5).

The previously proposed model [38] was developed to provide an intuitive framework for understanding how molecular parameters, such as uptake and leakage, shape community-level properties, including spatial arrangement and composition. To maintain analytical tractability, the model incorporated several simplifying assumptions: uptake was modeled using linear (non-saturating) kinetics, growth was treated as a death-birth process on a graph (neglecting the effects of radial expansion), and spatial structure was approximated using pairwise correlations [38]. Despite these simplifications, the model effectively captured key features of spatial organization and community composition observed in microfluidic experiments [38].

Colony range expansions, however, present a more complex scenario. Growth occurs in three dimensions, radial expansion induces pattern formation even in the absence of direct interactions, and molecule diffusion is influenced by both the densely packed colony and the underlying agar. Nevertheless, here we demonstrated that the model can be extended qualitatively and, to a degree, quantitatively to range expansion scenarios. The simplifying assumptions, although critical for analytical treatment, largely account for the model’s limitations, such as its inability to predict absolute sector size and its progressively reduced accuracy when overexpressing mutants are included. Although these assumptions could be relaxed by integrating elements from more comprehensive colony models [28, 47, 58], doing so would sacrifice analytical tractability, which remains a key advantage of this approach. Despite its simplicity, we experimentally tested and validated previously unconfirmed predictions of the model and in a 3D range expansion. In particular, we demonstrated that increasing uptake rates mainly affects spatial patterning, while increased leakage rates affect community composition. These findings underscore the model’s potential as a valuable tool for exploring how metabolite exchanges shape community-level dynamics.

In our image analysis, we focused on the edge of the range expansion, where the community grows and needs to exchange amino acids. At the edge, most cells grow outwards, where the nutrients are abundant and the space is free. This resembles the situation in a microfluidic chamber where the nutrients are only accessible from one side of the chamber and the cells typically grow in one direction only [59]. In contrast, in the center of a range expansion, the cells grow in all directions on the agar. However, they quickly merge with other microcolonies, which hinder their growth as they compete for space and nutrients. Typically, at the densities we worked with, the center was filled after 24 to 48 hours while the edge grew for up to five days. For future work, it would be interesting to study if the same rules also apply when growth occurs in all directions, as in the center of the range expansion experiments.

Do uptake and leakage rates also play a similar role for other auxotrophic communities? Preliminary results indicate that our observation might also hold true for other synthetic communities of amino acid auxotrophs. We tested a histidine auxotroph ($\Delta$*hisD*) and a methionine auxotroph ($\Delta$*metB*) in combination with $\Delta$*proC*. Again, we used *proB74* for proline overproduction and also built a histidine overproducer by deleting *hisL*, which had been shown to result in histidine overproduction by releasing the negative regulation of the pathway [57]. We were able to show that the frequency of the community members was changing according to the amino acid production level when proline or histidine were overproduced (Fig. S12). Similarly to proline and tryptophan, we have modified these auxotroph strains to overexpress specific importers for histidine and methionine (*hisJPQM* and *metNIQ*) [60, 61]. Although we detected differences in patterns, we could not quantify them with our current imaging and analysis pipeline, as the sectors are extremely small. We thus have some evidence that the same rules apply for other communities of amino acid auxotrophs, however, further research is required to see how general the uptake and leakage rates of exchanged metabolites determine spatial arrangement and composition of microbial communities.

Here, we studied synthetic communities in a controlled environment, this simple model system allowed us to understand principles that we believe are also relevant for natural situations and that would have been difficult to unravel in a more complex setting. Indeed, range expansions experiments like ours have been previously used to decipher ecological processes taking place in diverse microbial communities. These studies typically focused on known interactions within a community and described their effect on factors such as pattern formation, expansion radius or frequency of partners [31, 33, 34, 52, 62, 63]. Additionally, the initial conditions of the range expansion have been modified such as initial ratio or density of cells, resources availability or antibiotic stress [28, 51, 64–66]. Finally, genetic modifications have been applied to community members showing the effect of a specific feature of the cells on the final pattern, such as motility [53]. In our study, we investigated the effect of interactions between two strains that reciprocally exchange amino acids. These metabolites are also relevant in a variety of natural communities ([11, 16–19], even though their exchange is not necessarily mutual as in our engineered system. Moreover, we have shown that modifying the strength and range of metabolite exchanges by genetic modifications is a powerful approach to understand how they influence the microbial community. The parameters that we chose to modify, uptake and leakage rates, are also subject to change during evolution. For example, a recent laboratory evolution experiment with a microbial community of *E. coli* and *Saccharomyces cerevisiae* amino acid auxotrophs, selected mutations which led to increased uptake and production rates of the exchanged amino acids [67].

In addition to improving our understanding of how metabolite exchanges between community members determine community-level properties, the results that we describe here are also relevant for applications where the tight control of community composition is desirable [68]. For example, it is well established that distributing the bioproduction across a synthetic consortium can increase the yield compared to production in a single strain [69–71]. However, maintaining the stability of these engineered communities remains a challenge. Among other strategies, cross-feeding between community members has been used to establish stable communities [72, 73]. Similarly to the results presented here, we have observed that we can also control the frequency of members of our synthetic community in liquid cultures by changing leakage rates (Fig. S13). Modifying leakage and uptake rates might therefore be a promising approach to control the composition and spatial arrangement of synthetic co-cultures for bioproduction [74–77], but also for other applications such as food production [78], distributed biocomputing [79, 80] or engineered living materials [81].

## Supplementary Material

Supporting_information_wraf122

Source_Data_wraf122(1)

## Data Availability

The source data of experiments is provided in the Source Data file. All microscopy pictures are available on figshare: https://doi.org/10.6084/m9.figshare.26539699. Plasmids and strains are available at Addgene (see supplementary information for Addgene numbers).

## Code availability

The Python code used for model fitting and predictions is available on Zenodo: https://doi.org/10.5281/zenodo.12773415

The MATLAB code for image analysis is available on GitHub: https://github.com/SchaerliLab/Image_analysis_range_expansions.
