# Supplementary material for: Uptake and leakage rates differentially shape community arrangement and composition of microbial consortia
Source: ISME J. 2025 Jun 13;19(1):wraf122. doi: 10.1093/ismejo/wraf122 (PMC12422018; doi:10.1093/ismejo/wraf122)
Supplement: Supporting_information_wraf122 [file supporting_information_wraf122.pdf]

Supplementary information for
"Uptake and leakage rates differentially shape community arrangement and composition of microbial consortia"

Estelle Pignon[1], Gábor Holló[1], Théodora Steiner[1], Simon van Vliet[1,2], Yolanda Schaerli[1]

[1]Department of Fundamental Microbiology, University of Lausanne.
[2]Biozentrum, University of Basel.

Supplementary Figures

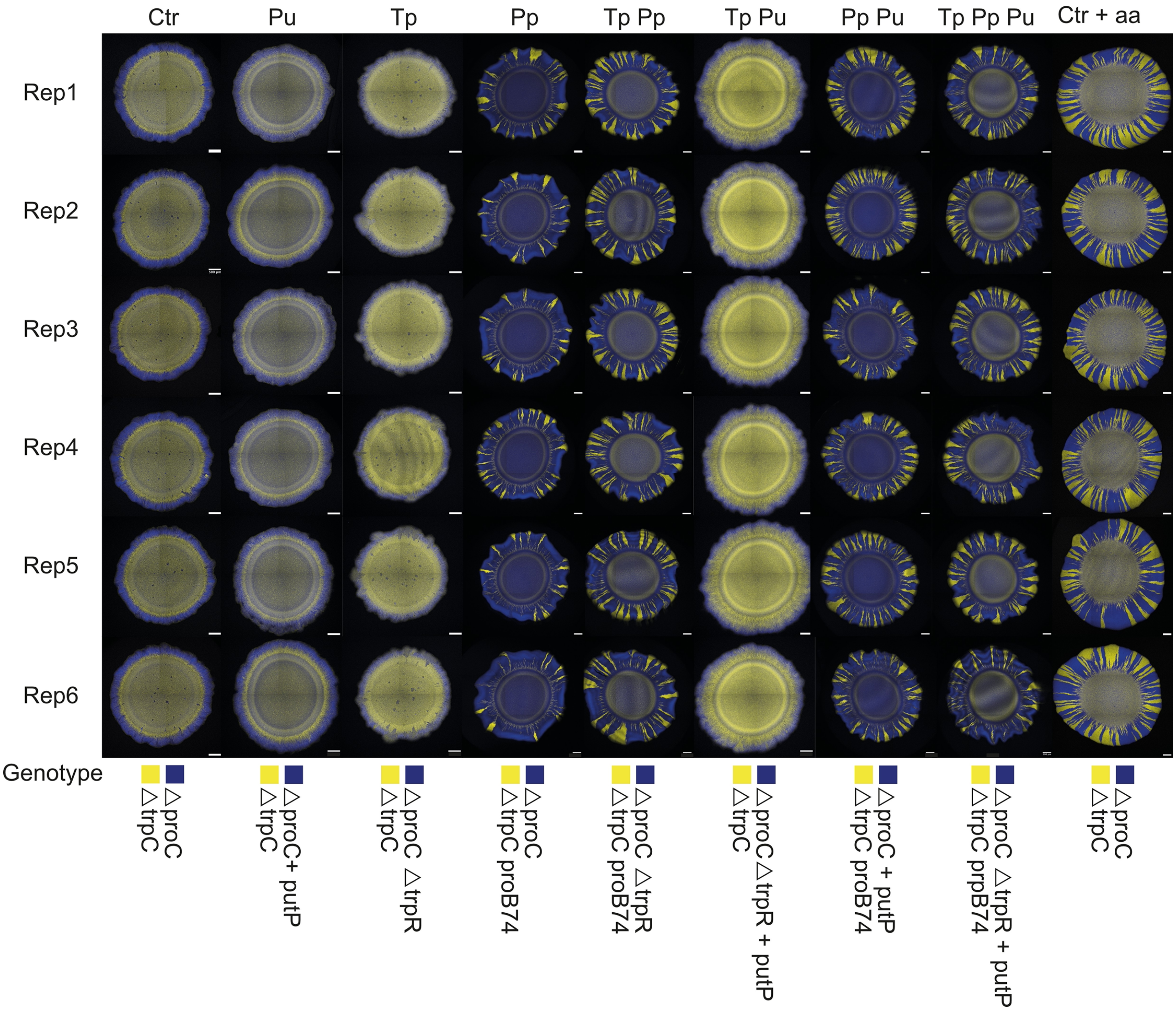

Figure S1: **Full images of the range expansions.** Full pictures of all 6 replicates for all communities analysed in this study. Scale bars = 500μm

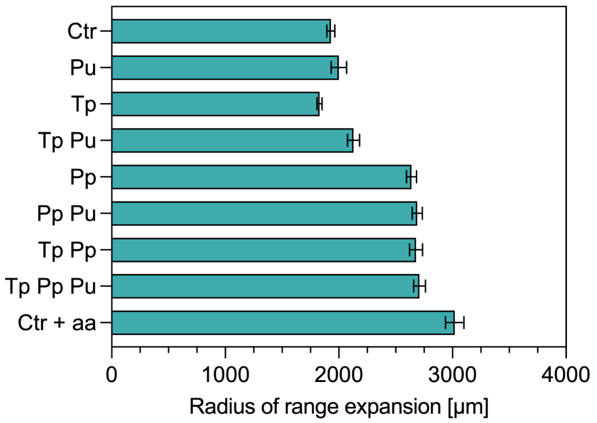

Figure S2: **Sizes of the range expansions**. Growth ranges in μm for all the different communities presented in this study. We subtracted the size of the initial inoculate at time 0 from the size of the final range expansion. Mean and standard deviation of 6 biological replicates.

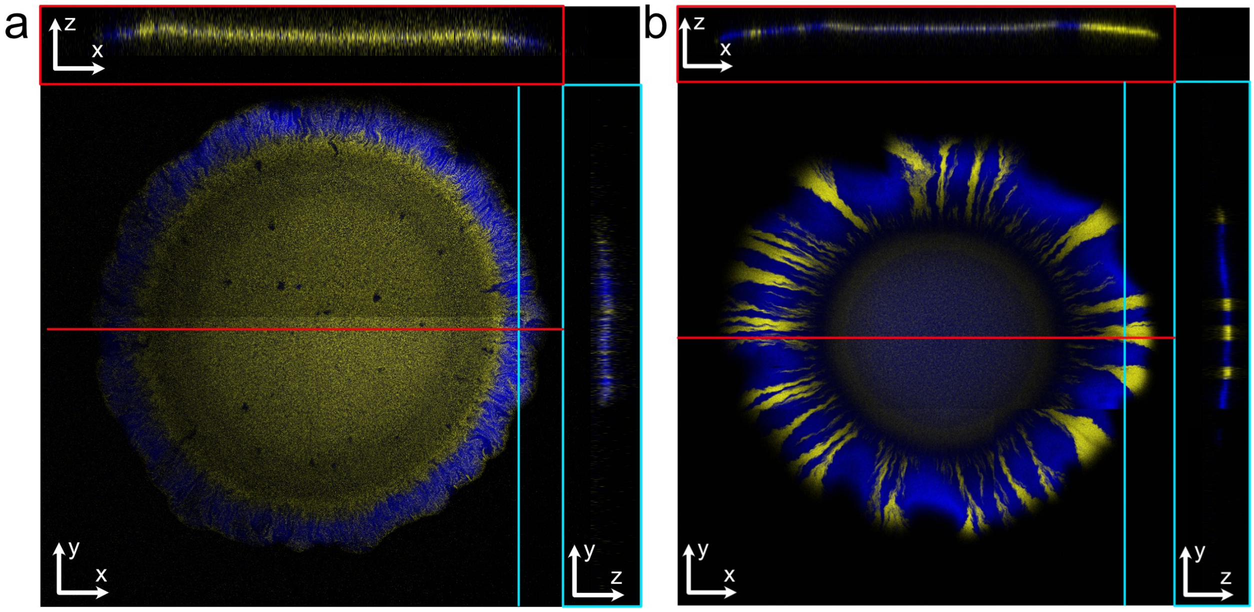

Figure S3: **Spatial patterns in the x, y, z dimensions for two representative communities. a.** Control (Ctr) community, with all side views. **b.** Proline overproduction and proline uptake (Pp Pu) community, with all side views.

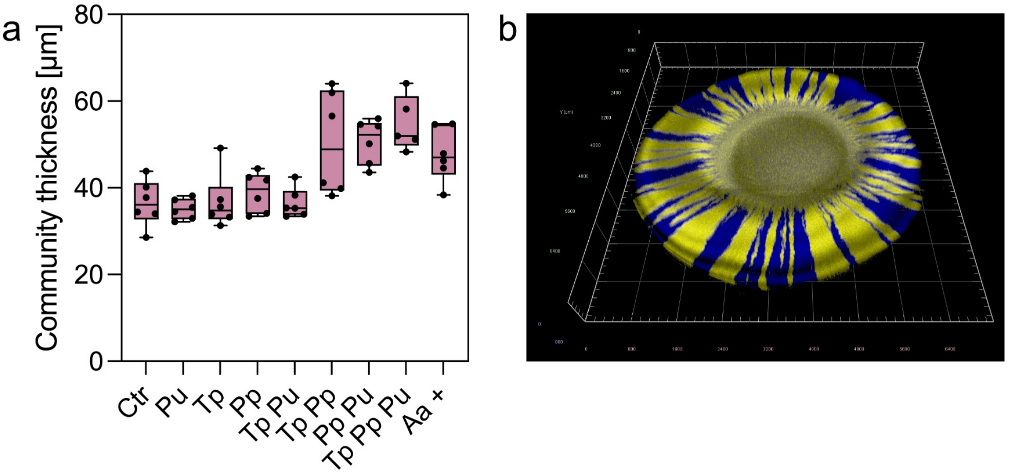

Figure S4: **Range expansions growing on agar plate in an unconstrained spatial setting.** The pictures that we analyse are a projection of multiple z-stack. **a.** Thickness of interval between first and last z-stack for each engineered community. **b.** Three dimensional representation of one range expansion (control community in the presence of proline and tryptophan as shown in Figure 1b of the main manuscript).)

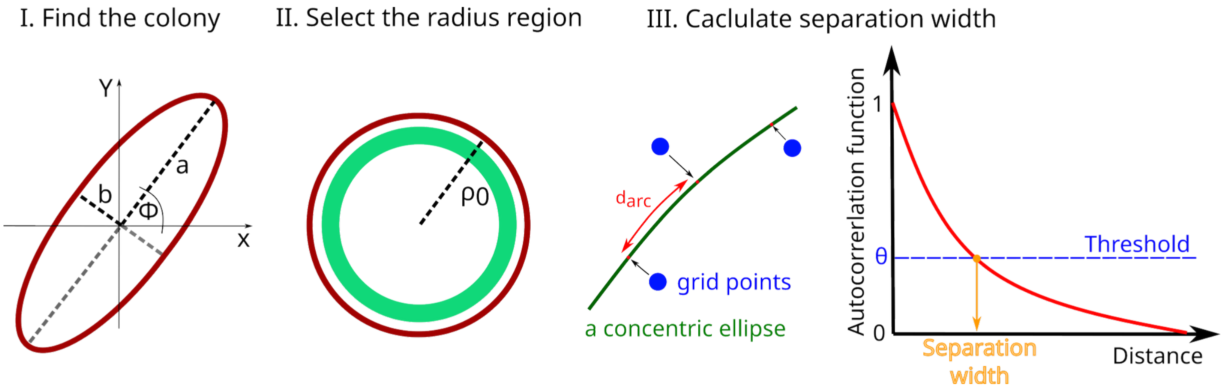

Figure S5: **The process of the image analysis. I.** The boundary of the colony is identified, and an ellipse is fitted to it. (Note: the ellipticity is greatly exaggerated in this sketch for clarity.) **II.** The ellipse is transformed into an equivalent circle, a specific radius range is selected, and the yellow ratio is calculated within this range. The radius of the equivalent circle ρ_0 is used to determine the range expansion. **III.** The arc length d_{arc} of the concentric ellipses is used to determine the distance between data points for the autocorrelation function. We then calculated the sector width based on the autocorrelation function, specifically identifying where its value falls below a predefined threshold (Θ). The impact of the selected threshold value is illustrated in Figure S13. Details of this process are provided in the image analysis section of the manuscript.

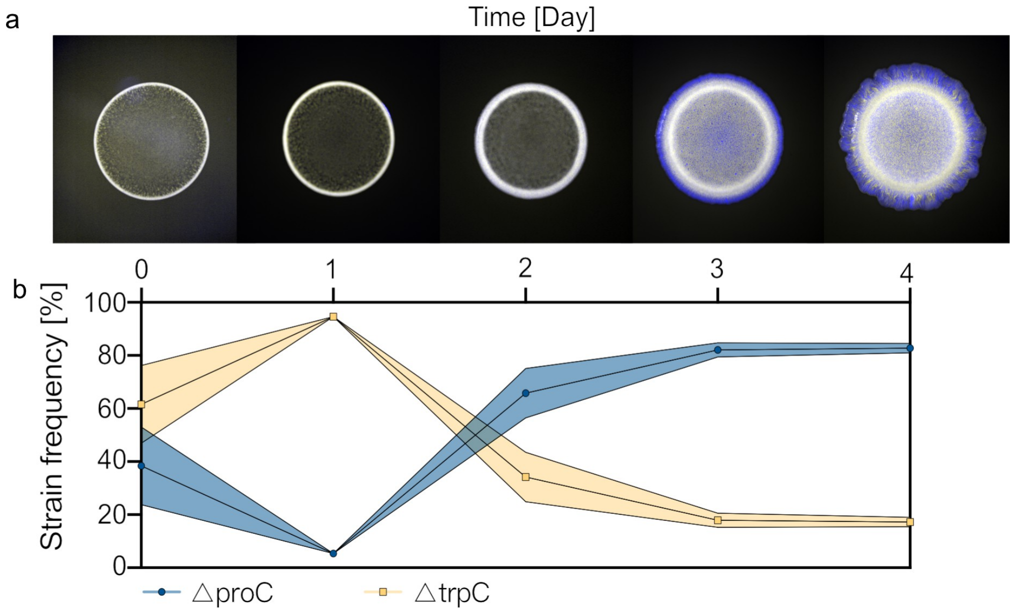

Figure S6: **Time lapse of control community without amino acids. a.** Microscopy images taken at 24h intervals. **b.** Quantification of strain frequencies over time with flow cytometry.

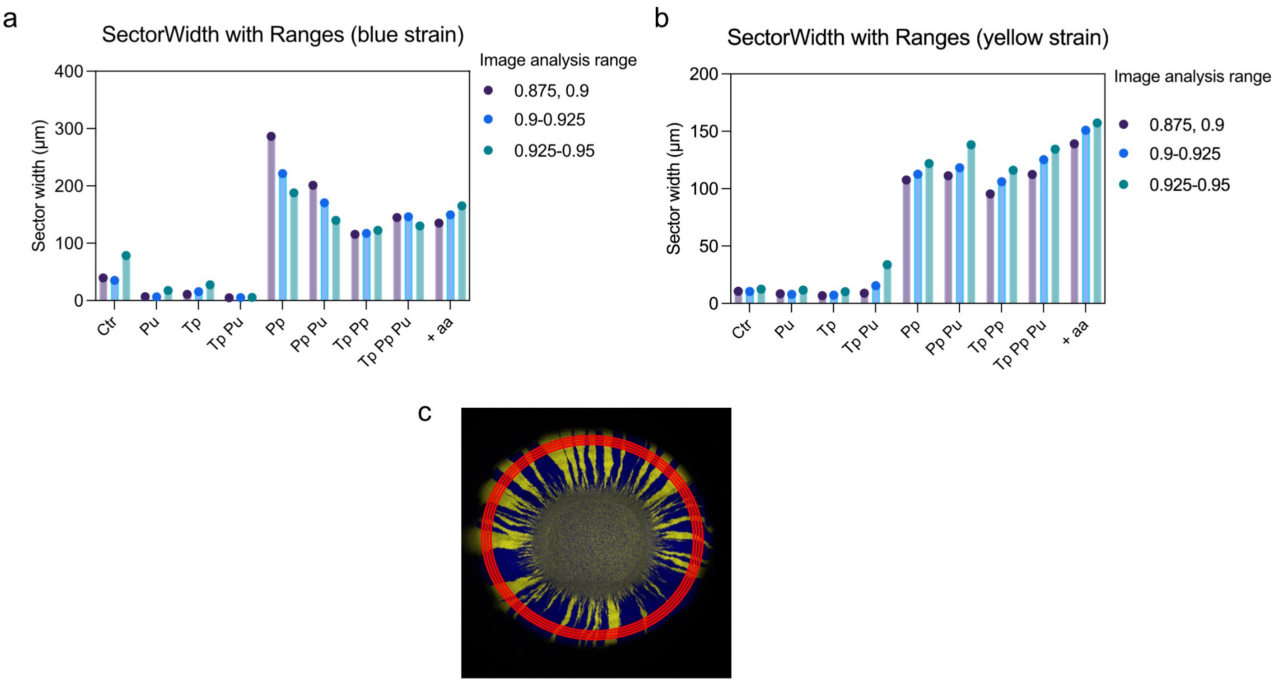

Figure S7: **Image analysis at different ranges of the radius, with center = 0 and edge = 1. a.** Sector width of all blue strains in different communities. **b.** Sector width of all yellow strains in different communities. **c.** Visual representation of the different ranges on a community (control community + amino acids).

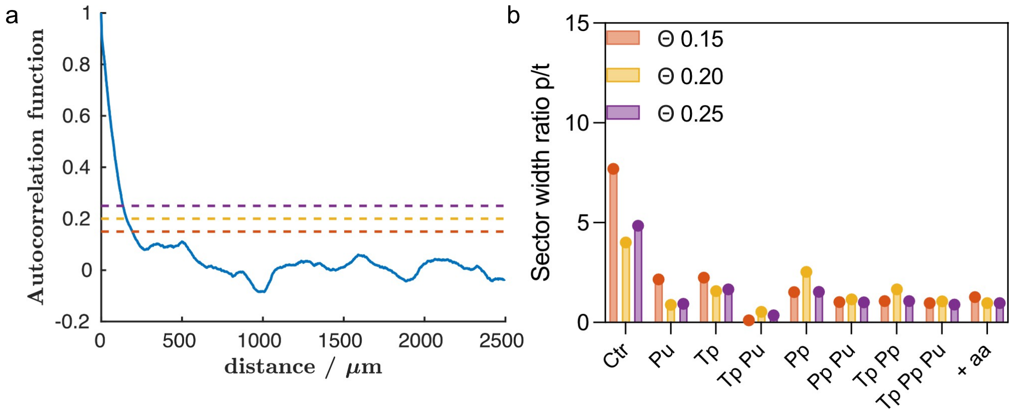

Figure S8: **Effect of different thresholds (theta) on image analysis results a.** Example of the autocorrelation function of the Ctr + amino acids community with three different thresholds (θ) (0.15-0.25) shown. **b.** Resulting values for sector width in each community for the three different threshold of the autocorrelation. We have used the threshold of 0.2 for all our image analyses.

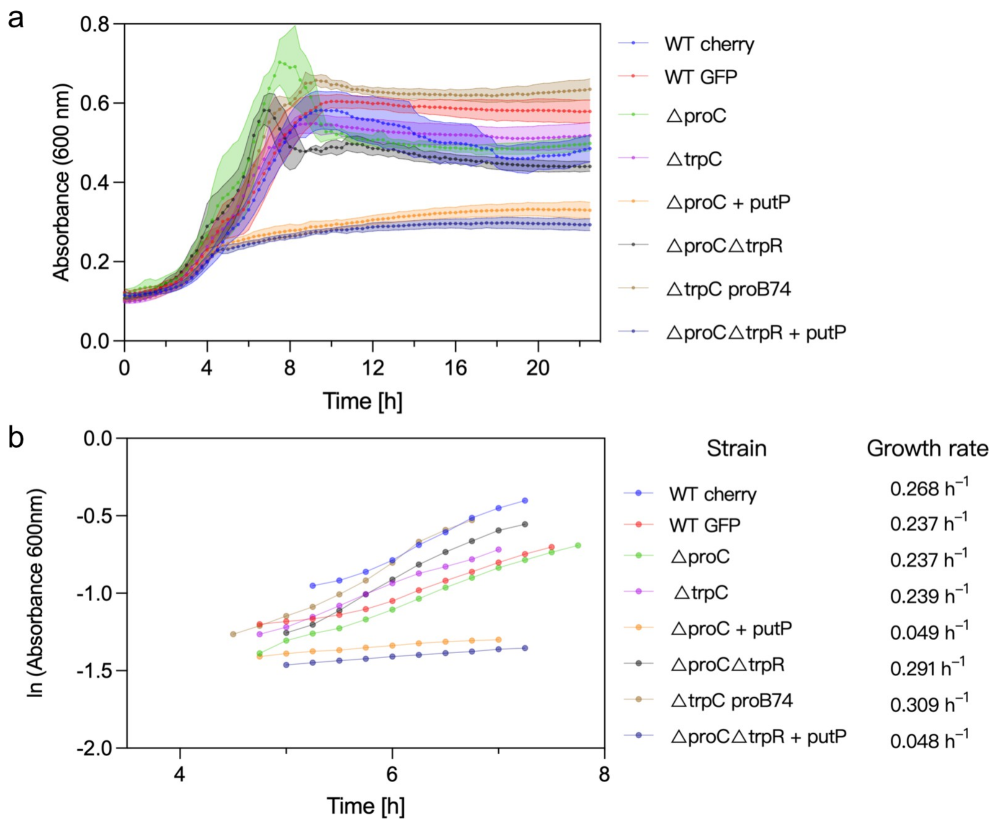

Figure S9: **Growth curves of all strains used in this study as mono-cultures.** Strains with indicated plasmids were grown in M9 liquid media supplemented with proline and tryptophan (50 mg/mL and 20 mg/mL). **a** Absorbance at 600 nm measured over 22 h with 15 min between time points. Mean and standard deviation for three biological replicates for each strain. **b** Growth rates. First, we plotted the natural logarithm of the absorbance data points (ln (Absorbance 600 nm)), then isolated the exponential growth phase of the curves by selecting the part of the curves that fitted the linear trendline with a R^2 higher than 0.9 and took the slope of this curve as growth rate (right column).

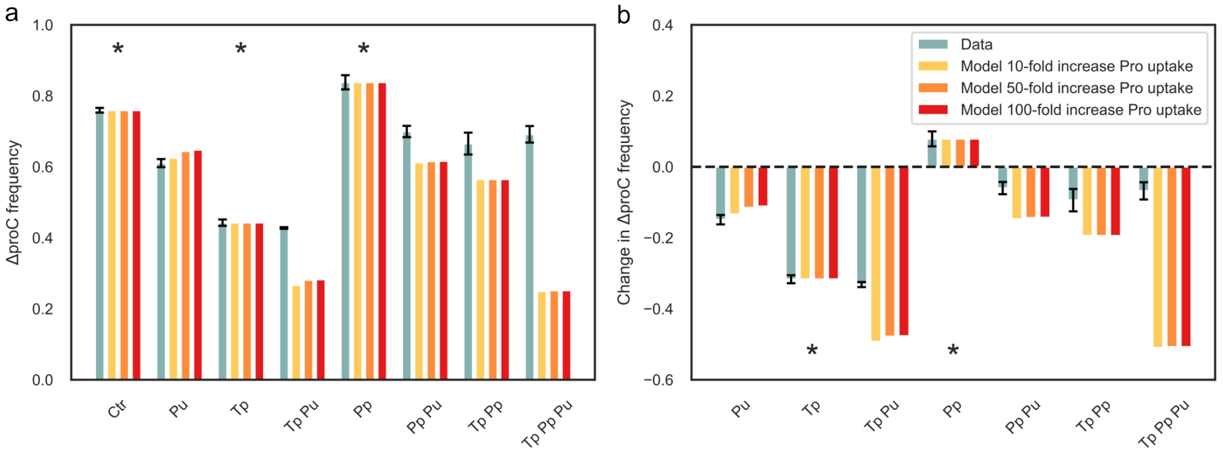

Figure S10: **Comparison between model predictions and experimental data for for strain frequencies**. **a** Comparison between the observed (blue) and predicted (yellow/orange/red) frequency of Δ*proC*. **b** Observed (blue) and predicted (yellow/orange/red) changes in the frequency of Δ*proC* relative to the Ctr community. Model predictions were made assuming three different values for the increase in proline uptake conferred by *putP* overexpression: 10-fold (yellow), 50-fold (orange), and 100-fold (red). For the data, mean values and standard errors are shown. ∗ indicate data that was used to fit the effective leakage rate of tryptophan and proline in the control and overexpressing strains.

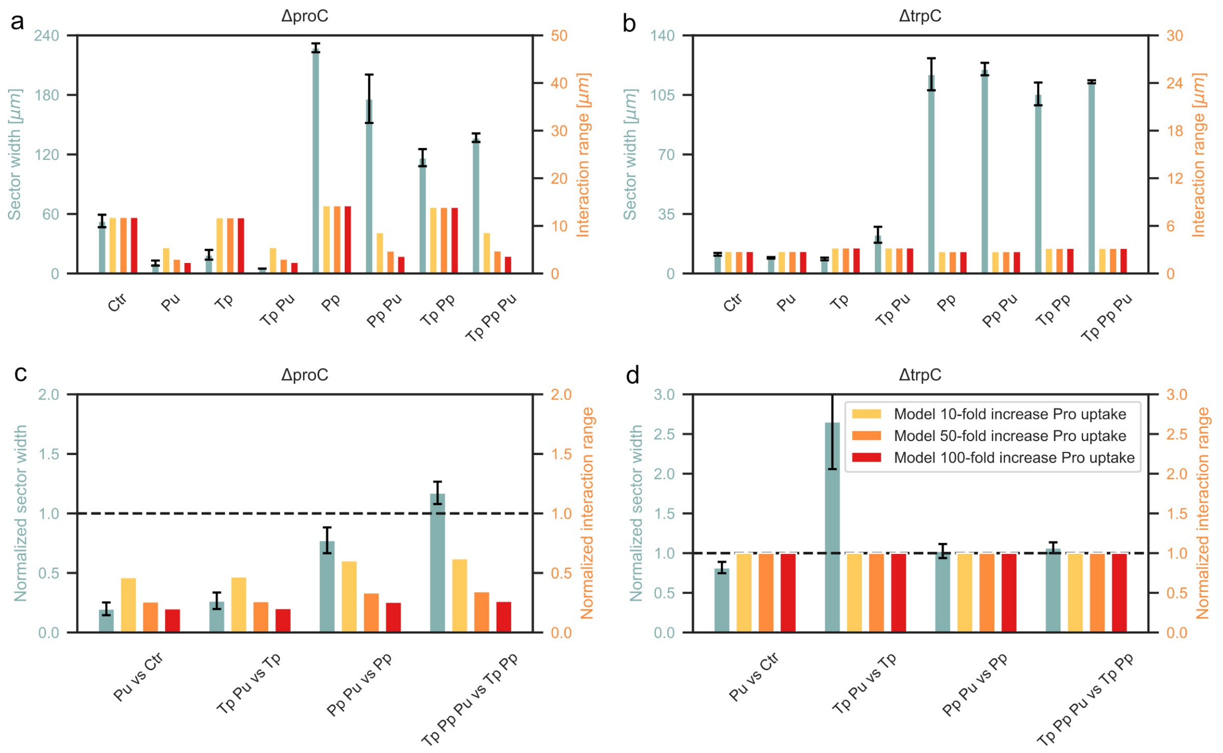

Figure S11: **Comparison between model predictions and experimental data for sector widths. a, b** Observed sector widths (blue, left-axis) and predicted interaction range (yellow/orange/red, right-axis) for Δ*proC* (**a**) and Δ*trpC* (**b**). Note that sector width and interaction range are not directly proportional, as sector width is also influenced by other—non-metabolic—factors. **c, d** The effect of increased uptake rate on sector width and interaction range was assessed by normalizing values in communities containing the *putP* -overexpressing strain (with increased proline uptake rate) to those in the corresponding communities without *putP*. Shown are normalized sector width (blue, left-axis) and normalized predicted interaction range (yellow/orange/red, right-axis) for Δ*proC* (**c**) and Δ*trpC* (**d**). Model predictions were made assuming three different values for the increase in proline uptake conferred by *putP* overexpression: 10-fold (yellow), 50-fold (orange), and 100-fold (red). For the data, mean values and standard errors are shown.

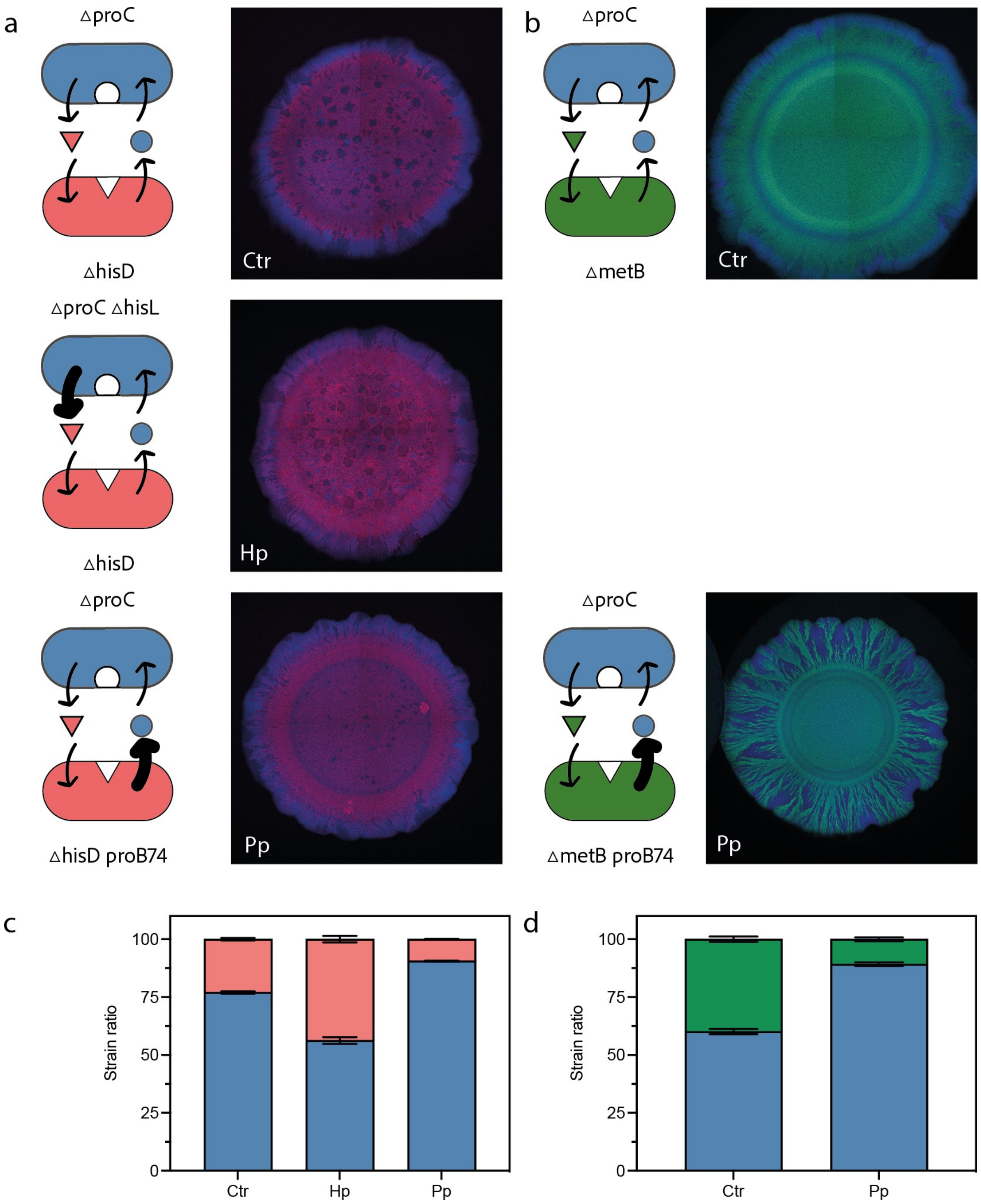

Figure S12: **Expanding to other amino acids auxotrophic strains a** Community of proline auxotroph and histidine auxotroph (Δ*hisD*). Control community (Ctr), histidine overproducer community (Hp) with Δ*hisL*, and proline overproducer community with proB74 mutant (Pp) . Representative microscopy images of the range expansion. **b** Community of proline auxotroph and methionine auxotroph (Δ*metB*). Control community (Ctr) and proline overproducing community with *proB74* mutant (Pp). **c** Quantification of the frequencies of the two members of the proline-histidine community. Data from 3 biological replicates. **d** Quantification of the frequencies of the two members of the proline-methionine community. Data from 3 biological replicates.

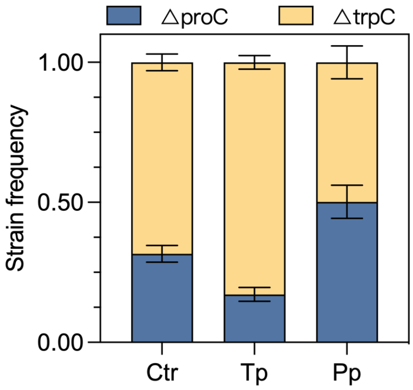

Figure S13: **Amino acid overproduction leads to a change of community composition in liquid cultures too.** Comparison between the initial community (Ctr) and communities with increased production of tryptophan (Tp) and proline (Pp) in liquid cultures (96 well plate)

Supplementary Methods

Effective Leakage Rate

The model of van Vliet et al. [1] was originally developed for densely packed microfluidic growth chambers and needed to be adapted to the range expansion setup where a densely packed colony grows on top of a large layer of agarose gel. This required an additional assumption: namely that the agar acts as an absorbing boundary: i.e. amino acids diffusing from the colony into the agar are lost from the system. To reflect the loss of amino-acids from the system, the leakage rate l has to be replaced with a lower effective leakage rate l_e. In this work we directly fitted the effective leakage rate to the data. However, here we illustrate the rationale of this approach using a simple diffusion model.

Consider a system where producers fully cover the $x-y$ plane and grow in a layer of height h. Producers release a flux of $l \cdot I_c$ [M/s] amino acids into the environment, which are taken up at a rate u [1/s]. These amino acids diffuse with an effective diffusion rate D_e [m²/s]. The concentration profile in the z-direction is then described by:

$$D_e \frac{d^2E(z)}{dt} = u \cdot E - l \cdot I_c \tag{1}$$

Now, consider two types of boundary conditions:

Growth Chambers

Here we have no-flux boundary conditions ($\frac{dE}{dz} = 0$) at both $z = 0$ and $z = h$, which gives as solution to Eq. 1:

$$E(z) = \langle E \rangle = \frac{l \cdot I_c}{u} \tag{2}$$

Colonies

Here we have a no-flux boundary condition ($\frac{dE}{dz} = 0$) at $z = h$ and an absorbing boundary condition ($E = 0$) at $z = 0$, which gives as solution to Eq. 1:

$$E(z) = \frac{l \cdot I_c \cdot (1 - \cosh[\frac{h-z}{r_0}] \cdot \text{sech}[\frac{h}{r_0}])}{u}$$

where $r_0 = \sqrt{D_e/u}$ [m]. Averaging $E(z)$ over $0 < z < h$ gives:

$$\langle E \rangle = \frac{l \cdot I_c}{u} \cdot (1 - \frac{r_0}{h} \cdot \tanh[\frac{h}{r_0}])$$

The average concentration of leaked amino acids is thus reduced compared to that in the closed chambers by a constant factor of $(1 - \frac{r_0}{h} \cdot \tanh[\frac{h}{r_0}])$. As a result, we can express the average concentration using the

same equation as for the growth chambers (Eq. 2) by replacing the leakage rate l with the effective leakage rate l_e:

$$\langle E \rangle = \frac{l_e \cdot I_c}{u} \tag{3}$$

with

$$l_e = l \cdot (1 - \frac{r_0}{h} \cdot \tanh[\frac{h}{r_0}])$$

Generally, we expect the colony height h in the growth zone at the colony edge to be smaller than the diffusion range r_0 (i.e. $h < r_0$), in which case the effective leakage rate l_e is significantly reduced compared to l.

Supplementary Tables

Table S1: **Strains used in this study**

**Strain name**	**Strain**	**Properties**	**Origin**	**Addgene ID**
ΔproC	ΔproC:FRT-mCherry:FRT	Auxotrophic for proline	[2]	230036
ΔtrpC	ΔtrpC:FRT-sfGFP	Auxotrophic for tryptophan	[2]	230037
ΔproCΔtrpR	ΔproC:FRT-mCherry:FRT ΔtrpR:FRT	Auxotrophic for proline and tryptophan overproducer	This study	229546
ΔtrpC proB74	ΔtrpC:FRT - proB D107A-sfGFP:FRT	Auxotrophic for tryptophan and proline overproducer	This study	229547
ΔhisD	ΔhisD:FRT-sfGFP	Auxotrophic for histidine	This study	229548
ΔmetB	ΔmetB:FRT - sfGFP:FRT	Auxotrophic for methionine	This study	229549
ΔhisD proB74	ΔhisD:FRT - proB D107A - sfGFP:FRT	Auxotrophic for histidine and proline overproducer	This study	229550
ΔmetB proB74	ΔmetB:FRT - proB D107A - sfGFP:FRT	Auxotrophic for methionine and proline overproducer	This study	229551
ΔproC ΔhisL	ΔproC:FRT - ΔhisL:FRT - mCherry:FRT	Auxotrophic for proline and histidine overproducer	This study	229552

Table S2: **Primers used in this study**

Primer name	Sequence (5'-3')	Description
prEP111	actagtagcggccgctgcagtc	Linearize pDSG360 plasmid (Forward
prEP112	ctctagtagtgctcagtatct	Linearize pDSG360 plasmid (Reverse)
prEP113	agatactgagcactactagagaaaga ggagaaatactagatggctattagcacacc	Amplify putP from MG1655 genome (Forward) homology to pDG360
prEP114	gactgcagcggccgctactagttaagt cccttagctttcctg	Amplify putP from MG1655 genome (Reverse) homology to pDSG360
prEP167	tacaaccgggggaggcattttgcttccc ccgctaacaatggcgacatatttgtag gctggagctgcttcg	Amplify FRT Kanamycin FRT from pDK3 with homology arms for deletion of trpR (Forward)
prEP168	gcattcggtgcacgatgcctgatgcgc cacgtcttatcaggcctacaaaagccatggtc-catatgaatatcctcc	Amplify FRT Kanamycin FRT from pDK3 with homology arms for deletion of trpR (Reverse)
prEP177	taattcccatgtcagccgttaagtg	Linearize pKD4 (Reverse)
prEP185	ttccatattagcacgggtcagcag	Amplify proB from MG1655 genome, includes a point mutation (A319G) on proB gene
prEP197	tcccgcgcaacaaaacgccatgctttg ctcgcagatggttggcaaccgatgtag gctggagctgcttcg	Amplify FRT Kanamycin FRT from pDK3 with homology arms for deletion of proBA (Forward)
prEP198	gtcaatggccttgtgaatcaaatggcta cttttgcatcacccggttttatgccatgg tccatatgaatatcctcc	Amplify FRT Kanamycin FRT from pDK3 with homology arms for deletion of proBA (Reverse)
prEP200	gccatggtccatatgaatatcctcc	Linearize pKD4 (Forward)
prEP201	ggaggatattcatatggaccatggccg acagtcctgctaaaacgtt	Amplify proB from MG1655 genome, homology to pKD4
prEP203	ctgctgacccgtgctaatatggaa	Amplify proBA from MG1655 genome, includes a point mutation (A319G) on proB gene
prEP205	ttcacgaacgtgaatcacggtggacaa ggttaaaactaaccgggcgatgcttta cgcacgaatggtgtaatcacc	Amplify FRT Kanamycin FRT and proBA (mutated) to integrate in genome
prEP206	ttcacgaacgtgaatcacggtggacaa ggttaaaactaaccgggcgatgcttta cgcacgaatggtgtaatcacc	Linearize pEP17, homology to pEP17, removing putP sequence
prEP207	gccagttcaattctggtcctgccgattg agaagatgatggagtgatcgcctgtag gctggagctgcttcg	Amplify FRT Km FRT with homology arms to hisD (F)
prEP208	cgtcaggttgcggacgttttcacgcgct aaatcggtaatagtcacggtgcgccat ggtccatatgaatatcctcc	Amplify FRT Km FRT with homology arms to hisD (R)
prEP211	ttactctggtgcctgacatttcaccgac aaagcccagggaacttcatcactgtag gctggagctgcttcg	Amplify FRT Km FRT with homology arms to metB (F)
prEP227	gtggtttaggttaaaagacatcagttga ataaacattcacagagactttttgtagg ctggagctgcttcg	Amplify FRT Km FRT with homology arms to hisL (F)
prEP228	atgcaccactggaagatctgaatgtctt ccagcacacatcgcctgaaagagcca tggtccatatgaatatcctcc	Amplify FRT Km FRT with homology arms to hisL (R)
prEP250	cattaaagaggagaaattaactatga gtgacagccagacgctg	Amplify FRT Kanamycin FRT and proBA (mutated)

Table S3: **Plasmids used in this study**

Plasmid name	Content	Origin	Addgene ID
pEP17	putP under ptet promoter - tetR - CmR	This study	229234
pEP28	empty plasmid - ptet and tetR - Cm resistance	This study	229235

Table S4: **Parameters used for model.** [a]: measured in units of the Monod constant. [b]: maximum growth rate in presence of amino acids, measured in batch cultures.

Parameter	Description	Strains	Value	Source
u_P	Uptake rate proline, WT	All but *putP*	2.04 $1/s$	[3]
c_{up}	Fold increase in uptake rate proline, *putP* $u_{P,putP} = c_{up} \cdot u_P$	*putP*	10-100	Free parameter
u_T	Uptake rate tryptophan, WT	All	24.05 $1/s$	[4]
l_P	Leakage rate proline, WT	All but *proB74*	$6.42 \cdot 10^{-7}$ $1/s$	Fitted
$l_{P,proB74}$	Leakage rate proline, *proB74*	*proB74*	$4.24 \cdot 10^{-6}$ $1/s$	Fitted
l_T	Leakage rate tryptophan, WT	All but Δ*trpR*	$4.71 \cdot 10^{-8}$ $1/s$	Fitted
$l_{T,trpR}$	Leakage rate tryptophan, Δ*trpR*	Δ*trpR*	$1.61 \cdot 10^{-6}$ $1/s$	Fitted
D_P	Diffusion constant proline	All	879 $\mu m^2/s$	[5]
D_T	Diffusion constant tryptophan	All	659 $\mu m^2/s$	[6]
I_C	Internal concentration produced amino acid[a]	All	20	[2]
ρ	Cell density	All	0.65	[2]
L	Cell length	All	5.2 μm	[1]
W	Cell diameter	All	0.68 μm	[1]
μ_n	Maximum growth rate[b]	ΔproC	0.24 $1/h$	Measured
μ_n	Maximum growth rate[b]	ΔproC *putP*	0.049 $1/h$	Measured
μ_n	Maximum growth rate[b]	ΔproC Δ*trpR*	0.29 $1/h$	Measured
μ_n	Maximum growth rate[b]	ΔproC *putP* Δ*trpR*	0.048 $1/h$	Measured
μ_n	Maximum growth rate[b]	ΔtrpC	0.24 $1/h$	Measured
μ_n	Maximum growth rate[b]	ΔtrpC *proB74*	0.31 $1/h$	Measured
μ_n	Maximum growth rate[b]	WT	0.27 $1/h$	Measured

References

1. Vliet, S. v., Hauert, C., Fridberg, K., Ackermann, M. & Co, A. D. Global dynamics of microbial communities emerge from local interaction rules. *PLOS Computational Biology* **18,** e1009877 (2022).
2. Dal Co, A., van Vliet, S., Kiviet, D. J., Schlegel, S. & Ackermann, M. Short-range interactions govern the dynamics and functions of microbial communities. *Nature Ecology & Evolution* **4,** 366–375 (2020).
3. Grothe, S., Krogsrud, R. L., McClellan, D. J., Milner, J. L. & Wood, J. M. Proline transport and osmotic stress response in Escherichia coli K-12. *Journal of Bacteriology* **166,** 253–259 (1986).
4. Piperno, J. R. & Oxender, D. L. Amino acid transport systems in Escherichia coli K-12. *The Journal of Biological Chemistry* **243,** 5914–5920 (1968).
5. Wu, Y., Ma, P., Liu, Y. & Li, S. Diffusion coefficients of l-proline, l-threonine and l-arginine in aqueous solutions at 25°C. *Fluid Phase Equilibria* **186,** 27–38 (2001).
6. Longsworth, L. G. Diffusion Measurements, at 25°, of Aqueous Solutions of Amino Acids, Peptides and Sugars. *Journal of the American Chemical Society* **75,** 5705–5709 (1953).
